# A novel functionalized CuTi hybrid nanocomposites: facile one-pot mycosynthesis, characterization, antimicrobial, antibiofilm, antifouling and wastewater disinfection performance

**DOI:** 10.1186/s12934-024-02400-6

**Published:** 2024-05-23

**Authors:** Asmaa G. Almahdy, Ahmed El-Sayed, Marwa Eltarahony

**Affiliations:** 1 Botany and Microbiology Department, Faculty of science, Damietta University, Damietta, Egypt; 2 Environmental Biotechnology Department, Genetic Engineering and Biotechnology Research Institute (GEBRI), City of Scientific Research and Technological Applications (SRTA-City), New Borg El- Arab City, Alexandria 21934 Egypt

## Abstract

**Background:**

The continuous progress in nanotechnology is rapid and extensive with overwhelming futuristic aspects. Through modernizing inventive synthesis protocols, a paradigm leapfrogging in novelties and findings are channeled toward fostering human health and sustaining the surrounding environment. Owing to the overpricing and jeopardy of physicochemical synthesizing approaches, the quest for ecologically adequate schemes is incontestable. By developing environmentally friendly strategies, mycosynthesis of nanocomposites has been alluring.

**Results:**

Herein, a novel architecture of binary CuO and TiO_2_ in nanocomposites form was fabricated using bionanofactory *Candida sp.*, for the first time. For accentuating the structural properties of CuTi nanocomposites (CuTiNCs), various characterization techniques were employed. UV-Vis spectroscopy detected SPR at 350 nm, and XRD ascertained the crystalline nature of a hybrid system. However, absorption peaks at 8, 4.5, and 0.5 keV confirmed the presence of Cu, Ti and oxygen, respectively, in an undefined assemblage of polygonal-spheres of 15–75 nm aggregated in the fungal matrix of biomolecules as revealed by EDX, SEM and TEM. However, FTIR, ζ-potential and TGA reflected long-term stability (− 27.7 mV) of self-functionalized CuTiNCs. Interestingly, a considerable and significant biocide performance was detected at 50 µg/mL of CuTiNCs against some human and plant pathogens, compared to monometallic counterparts. Further, CuTiNCs (200 µg/mL) ceased significantly the development of *Staphylococcus aureus*, *Pseudomonas aeruginosa* and *Candida albicans* biofilms by 80.3 ± 1.4, 68.7 ± 3.0 and 55.7 ± 3.0%, respectively. Whereas, 64.63 ± 3.5 and 89.82 ± 4.3% antimicrofouling potentiality was recorded for 100 and 200 µg/ml of CuTiNCs, respectively; highlighting their destructive effect against marine microfoulers cells and decaying of their extracellular polymeric skeleton as visualized by SEM. Moreover, CuTiNCs (100 and 200 µg/ml) exerted significantly outstanding disinfection potency within 2 h by reducing the microbial load (i.e., total plate count, mold & yeast, total coliforms and faecal *Streptococcus*) in domestic and agricultural effluents reached >50%.

**Conclusion:**

The synergistic efficiency provided by CuNPs and TiNPs in mycofunctionalized CuTiNCs boosted its recruitment as antiphytopathogenic, antibiofilm, antimicrofouling and disinfectant agent in various realms.

## Background

Despite the all Earth’s spheres are occupied with microbial populations that exert their best in serving ecosystem beneficially, other indigenous indwellers are also pathogenic. Even though some pathogens don’t injure humans directly, they are soil-borne, water-borne and air-borne pathogens, and their perils thereupon touch near or far human health, the ambient ecosystem and even the economy [1]. Soil-borne pathogens or phytopathogens, including bacteria, fungi, viruses, protozoa and nematodes, are widely disseminated in soil, causing lethal alterations in all plant growth phases, post-harvest and throughout storage. Thereby, any phytopathogenic infection would influence adversely fruits/vegetables’ nutritional value, organoleptic quality, half-life and subsequently devastation in crops followed by greater economic losses. Let alone the ability of phytopathogens to produce mycotoxins and other fungal metabolites that directly defeat human health by causing teratogenicity, carcinogenicity, hepatotoxicity, nephrotoxicity, reproductive disorders and immunosuppression [2].

In the same context, inadequate irrigation practices, infested farm animal feces and poor human handling are the substantial reasons for food-borne microbial contamination throughout the whole agricultural and subsequent industrial stages till practical consumption. Similarly, the improper water disinfection procedures and unintended mixing of sewage with drinking water pipelines and groundwater generate water-borne diseases [3]. Worthwhile, the pathogenic microbes could exist as planktonic (free-floating) or sessile (biofilm) phases. Via such later prophylactic mode (i.e., biofilm), the cells pose a superior resistance to biocidal agents and environmental stressors; consequently, more complications would face all stages of agriculture, the food processing industry, water purification plants and marine industry, as well as medical fields, where the biofilms eradication become a necessity, else while heavy benignity and economic losses would be recorded [4, 5].

Irrespective of the particular contamination pathways, taxonomic identities, or varieties involved, microbial pollution can eventually become bioavailable through food chains resulting in dietary exposure and exerting undesirable detrimental impacts on human health and socioeconomics. Hereby, the implementation of proper management strategies and effective antagonistic measures is critically important for safeguarding food, water, and soil quality against pathogen contamination. Conventionally, a diverse array of synthetic biocidal agents and disinfectants, including azoles, chlorine, chlorine dioxide, chloramines, aldehydes, phenols, esters, organic acids, isothiazolinones, halogens, and oxidizing agents, have been utilized to suppress the virulence across a broad spectrum of pathogens, as substantiated through extensive evidence of inhibitory efficacy across myriad application scenarios [2, 6]. However, their residues or even their byproducts on plants, fruits, and water and also their long-term persistence elicits profound concerns regarding human health, environmental impact and ultimately economic burdens. Remarkably, the extensive and abuse of synthetic biocide compounds can induce selective pressure in pathogens, facilitating mutations, which ending with biocides tolerance phenomena ; thereby escalating the hazard [7]. Thus, finding of efficient alternatives that are cost-effective, environmentally benign with an extensive public acceptance is prerequisite from international environmental agencies [2].

The recent progress in nanotechnology has been rapid and extensive with overwhelming futuristic aspects by modernizing inventive synthesis protocols and characterization techniques [8, 9]. The application of engineered materials, especially metals and metal oxides, in their nanoscale is regarded as one of the most recent promising solutions for challenging microbial pollution and ceasing the dissemination of multidrug-resistant microorganisms (MDR) [10]. The enhanced properties (i.e. catalytic, magnetic, optical, thermal, biological, etc.) of nanostructures promote technologists to develop distinct formulations, which find their avenue in diverse realms of plant disease management, skin care products, food packaging/preservatives, antitumor agents, drug delivery, and water disinfection [11–13]. The miniature dimensions and augmented surface-area-to-volume ratios of metals/metal oxide nanoparticles grant an enhanced ability to penetrate microbial cells, inflicting multi-modal antimicrobial mechanisms that impede the rapid evolution of target-specific resistance [14]. Remarkably, the strategic integration of geometrically diverse nanomaterials into unified composite nanoarchitectures yields a collective choir of complementary properties. This synergistic phenomenon dramatically augments the overall functional capabilities and mitigating the limitations of individual nanoparticle varieties. Accordingly, these multifunctional nanocomposite formulations engender manifold impactful advances across an extensive spectrum of technical disciplines and commercial sectors [11, 15, 16]. Noteworthy mentioning the fruitful multifaceted applications of metallic nanocomposites in water disinfection [17], dye degradation [18], phenols, and heavy metal detoxication [19, 20], self-healing [20], bio-blocks [21], bio-cement [22, 23], energy storage [24] and bio-implants [25].

Different studies reported the efficacy of amalgamation between CuO and TiO_2_ in their nanocomposites (NCs) form; assigning such efficiency to elevated synergistic activity and their quantum confinement effects, which consequently improved antimicrobial and photocatalytic performance, relative to their sol counterparts of CuNPs and TiNPs [26–28], and [29]. Additionally, their low price, higher compatibility, anti-toxicity, chemical stability, low band gap (Cu, 1.208 eV p-type; Ti, 3.2 eV: 3.6 eV n-type) and the whole multi-functionality reinforced their application in food nano-packaging, bioapplications, antimicrobial photodynamic/ photo-catalytic therapeutic and photothermal disinfection [30–32], and [21]. Presently, the incorporation of one of their oxides in nanocomposite hybrids was agreed by the FDA for vast domestic application in drugs, cosmetics, food, and food packaging industries as it can lessen or eradicate their toxic impacts [32–34], and [35]. Remarkably, almost all published scholars attributable to such bifunctional systems of CuNPs and TiNPs were prepared via physicochemical means such as spray pyrolysis, electro-spinning and chemical reduction [26–28], and [29]. The biogenic synthesis route of their nanocomposites (NCs), in particular microbial approach, is highly limited or not reported. It is plausible to mention the merits of biogenic methods symbolizing in their biocompatibility, environmental safety, cost-effective and overlook the utilization of temperature, energy, pressure, complicated equipment and toxic chemicals and residues [36, 37].

As a biogenic tool, yeasts occupy a prominent position as mediators for synthesizing non-metallic, metallic (alloy) and metal oxides NPs either intracellularly or extracellularly [38–40]. Let alone their primary and secondary metabolites, which are the hallmarks of metabolic engineering and synthetic biology in food and biopharmaceutical industries [41]. However, their easy-manipulative culturing, elevated metal bioaccumulation potentials, larger biomass with higher yields of metabolites per biomass unit, higher adaptability, diverse detoxication mechanisms and higher tolerance to suppressors positioned them as a platform for novel biotechnological applications [42, 43].

In light of the aforementioned, this study focused on mycofabrication of functionalized CuNPs, TiNPs and their –based nanocomposites (CuTiNCs) in the simultaneous existence of Cu and Ti salts. The structural properties of CuNPs, TiNPs and CuTiNCs were scrutinized using physicochemical characterization techniques of the transmission electron microscope (TEM), scanning electron microscope (SEM), X-ray diffraction (XRD), The Energy Dispersive X-ray microanalysis (EDX), UV-Vis spectrophotometry, Fourier transform infrared (FTIR), zeta potential and thermal gravimetric analysis (TGA). Thereafter, the antimicrobial capability of CuTiNCs was determined comparatively with the individual CuNPs and TiNPs; in addition, the behavior of CuTiNCs in prohibiting biofilm development and microfouler were evaluated. Moreover, the disinfection strength in eradicating the microbial load (bacteria, fungi and indicator organisms) in real environmental samples was assessed as well. Interestingly, no investigation to the best of the authors’ acquaintance has so far been published concerning microbial synthesis of CuTi hybrid, in particular by yeast isolate.

## Materials and methods

### Materials

The chemicals and media utilized in the current study were: DeMan-Rogosa agar (MRS agar) (OXOID), LB broth (Sigma Aldrich), Mueller Hinton (MH) agar (Sigma Aldrich), Trypticase Soy Broth (TSB) (Sigma Aldrich), Plate Count agar (PCA) (Sigma Aldrich), Rose Bengal chloramphenicol (RBCA), (Himedia), Violet Red Bile Agar (VRBA) (Sigma Aldrich), m-Enterococcus agar (Sigma Aldrich), Cu (NO_3_)_2_ (Sigma Aldrich) and C_12_H_28_O_4_Ti (Sigma Aldrich).

### Methods

#### Collection of samples, screening and cultural conditions

Several raw milk samples (from cows and buffalos) locally produced in Damietta governorate, Egypt were collected, evenly mixed, preserved in sterile bottles and placed in ice until arrival to the laboratory. As the samples were dairy products, the screening process for microbial nanofactories, to fabricate both nanoparticles and nanocomposites (NPs-NCs), were performed using serial dilution method on DeMan-Rogosa agar (MRS) agar, with the following ingredients (g/L): 10.0 Peptone, 5.0 Meat extract, 5.0 Yeast extract, 20.0 D (+)-Glucose, 2.0 K_2_HPO_4_, 2.0 Na_2_C_6_H_6_O_7_, 5.0 CH_3_COONa, 0.1 MgSO_4_, 0.05 MnSO_4_ and 15 agar, pH 6.5 ± 0.2, supplemented with 1.5 mM of NPs-precursors (i.e., Cu (NO_3_)_2_, and C_12_H_28_O_4_Ti). After incubation for 5 days at 30 °C, the isolate exhibiting color-changing (dark brown) was selected and considered as NPs-NCs producer [44]. The selected isolate underwent morphological, cultural and microscopic characterization. Actually, it exhibited the highest performance in NPs-NCs synthesis on MRS broth, thence, the cultural features were determined by aerobic and anaerobic incubation on MRS. Besides, its capability to grow on different pH ranges was examined by adjusting initial MRS broth in the range of 3–10. Moreover, the freshly prepared cultures were incubated at 4 °C, 10 °C, 20 °C, 30 °C, 40 °C, 50 °C, 60 and 70 °C to deduce its thermal tolerance. The morphology and dimensions of the selected strain was visualized by photomicrographs utilizing scanning electron microscopy (SEM) (JEOL JEM-1230, Japan) [44]. Regarding the molecular characterization, it was implemented by amplifying ITS region by using ITS-primer sets of (ITS1, 5′-TCCGTAGGTGAACCTGCGG-3′) and (ITS4, 5′TCCTCCGCTTATTGATATGC-3′). The obtained amplicon (∼ 600-base pair (bp) DNA) fragment was sequenced by an ABI 3730 automated sequencer (PerkinElmer/Applied Biosystems (Foster City, CA, USA). The BLASTn analysis was employed to assess the similarity, and its corresponding accession number was inquired; the phylogenetic tree was constructed by the MEGA- 6 software package via the neighbor-joining (NJ) method with bootstrap analyses for 1000 replicates [44].

#### Mycosynthesis approach of NPs-NCs

For detecting intracellular synthesis of NPs-NCs, a yeast lawn of 0.5 McFarland (≈ 10^8^ CFU/ml), taken from a previous freshly prepared culture, was inoculated in 250 mL Erlenmeyer flasks containing 70 mL of (MRS broth) (Oxoid) supplemented with 1.5 mM of (i.e., Cu (NO_3_)_2_, 1.5 mM of C_12_H_28_O_4_Ti and both. The precursors’ concentrations were selected based on the minimal inhibitory concentration (MIC) test (data not shown) [45]. However, the extracellular synthesis approach was monitored by inoculating cell-free filtrate of fresh culture (100 mL) with the exact concentrations of metals’ precursors either solely or mixed. All flasks were incubated for 96 h at 30 °C under orbitally shaking conditions (150 rpm). Concurrently, control flasks, namely, MRS broth containing yeast culture without metal precursors and MRS broth containing metal precursors without yeast cultures, were run in parallel with the test flasks. The primary sign of NPs-NCs fabrication was monitored visually throughout the incubation period via color changes in media and biomass from pale yellow to white, dark brown and whitish brown; indicating a successful formation of TiNPs, CuNPs and CuTiNCs, respectively [44, 45]. After complete incubation, the precipitates containing NPs-NCs either from intracellular or extracellular test flasks were harvested by centrifugation at 12,000×g for 20 min. The harvested pellets were rinsed thrice with distilled H_2_O to eliminate any residues, thereafter, the pellets containing NPs were washed and dried in oven (70 °C for 2 h) for subsequent characterization and application stages.

### Characterization of the mycosynthesized NPs-NCs

A number of different experimental techniques were used to obtain characteristic details on NPs-NCs in the terms of optical, morphology, size, chemical composition, phase identity, thermal, surface charge and functional properties. The optical properties of the NPs-NCs were first determined at room temperature using UV– Visible spectrophotometer, (Labomed model-double beam) within a wavelength range of 200–800 nm to detect Surface Plasmon Resonance (SPR). For the morphological properties and size, transmission electron microscopy (TEM) [JEOL JEM-1230-Japan], operated at 200 kV and Scanning electron microscopy (SEM) (JEOL JSM-6360LA) were employed [45]. For analyzing the physical configuration, phase identification and crystallinity of NPs-NCs, X-ray diffractometer (Shimadzu 7000, USA) operated with Cu Kα radiation (λ = 0.15406 nm), generated at 30 kV and 30 mA with a scan rate of 2°/min for 2θ values across a wide range of Bragg angles 10° ≤ 2θ ≤ 100, was employed in X-ray diffraction (XRD) analysis. However, the elemental components of the examined NPs-NCs were detected qualitatively and quantitatively by energy dispersive X-ray (EDX) detector connected with SEM- JEOL, JEM-1230- Japan [44]. Dynamic light scattering (DLS) technique was employed using Zetasizer Nano-ZS (Malvern Instruments, Worcestershire, UK) for measuring particle size distribution and zeta potential (ζ-potential), which reflect the colloidal stability of NPs and, in turn, to indirectly assess the surface charge and electrostatic potential [46]. The measurements were carried out at 25 °C, at a fixed scatter angle of 173° and the results were processed using Zetasizer software. The thermal properties were conducted by a TGA analyzer (TGA-50 H, Shimadzu, Japan), in a nitrogen atmosphere at a temperature range of 35–1000 °C and with a heating rate of 20 °C /min [47]. However, Shimadzu FTIR-8400 S, Japan, was used to analyze the functional groups associated with NPs-NCs. The spectrums of FTIR were measured at a spatial resolution of 4 cm^− 1^ in the transmission mode, between 4700 and 400 cm^− 1^ employing the disc technique [46].

### Applications of mycosynthesized NPs-NCs

#### Antimicrobial potency

The antagonistic potentiality of mycosynthesized CuNPs, TiNPs and CuTi-NCs were estimated by well diffusion method [28, 48]. Different pathogens from different microbial categories were examined including, human pathogens [*Bacillus cereus* (ATCC 33,019), *Staphylococcus aureus* (ATCC 29,213), *Pseudomonas aeruginosa* (ATCC 15,442)] and yeast [*Candida albicans* (ATCC 10,231)] and also plant pathogens [*Erwinia carotovora, Erwinia amylovora, Pseudomonas solanine, Pseudomonas syringae, Pedobacter carotovorum, Xanthomonas oryzae, Xanthomonas campestris, Agrobacterium tumefaciens Ralstonia solanacearum* and *clavibacter michiganensis*]. Such phytopathogenic microbes were isolated, identified and procured from the Plant Pathology Department, Faculty of Agriculture, Alexandria University. In brief, a single colony of each examined pathogen was inoculated in LB broth and incubated for 24 h at 30 °C on a rotary shaker at 150 rpm. A lawn of culture from each pathogen was prepared by spreading 100 µL of fresh culture containing 10^6^ CFU/mL on Mueller Hinton (MH) agar plates (Peptone 17.5, Meat extract 2.0, starch 1.5, agar, 17.0 g/L, pH 7). The inoculated plates were left standing for 10 min to let the culture get absorbed. Then 3 wells/ plate each of 8 mm in diameter were punched into the MH agar plates using sterile gel puncher (cork borer) and loaded with 50 µg/mL of CuNPs, TiNPs, and CuTiNCs. The plates were incubated at 35 °C ± 2 for bacteria and 25 °C ± 2 for yeast. After 24 h incubation period, the plates were investigated for the presence of zone of inhibition (ZOI), which was measured by subtracting the well diameter from the total inhibition zone diameter and expressed in centimeters (cm) [48, 49]. All tests and control samples were run in triplicates and the results were expressed as mean ± standard error of mean (SEM).

#### Antibiofilm activity

The biocidal function of TiNPs, CuNPs and their composites were investigated against biofilm-producing *S. aureus*, *P. aeruginosa* and *Candida albicans* cultures. A Sterile 96-well polystyrene microtiter plate well was inoculated with 10 µl of each culture solution adjusted to an OD_600_ of 0.1 (0.5 McFarland standard) in 100 µl of Trypticase Soy Broth (TSB) supplemented with 1% w/v glucose, followed by the addition of NPs-NCs (50,100 and 200 µg/ml). Besides, two controls; (positive control wells: medium containing bacterial suspension and negative control wells: containing just the sterile medium) were loaded in parallel. The microtiter plates were sealed and incubated at 37 °C for 24 h under stationary conditions to allow the biofilm development. Afterwards, the well content was drained, rinsed, and the residual biofilm was fixed and stained using 95% ethanol and 0.25% crystal violet, respectively. Using a plate reader (Tecan Infinite M200, Switzerland), the absorbance of the attached cells-dye mixture was spectrophotometrically measured at 595 nm, and the biofilm inhibitory percentage was estimated by Eq. (1). All tests and control samples were run in triplicates and the results were expressed as mean ± SEM [50, 51].

1 $$\mathbf{T}\mathbf{h}\mathbf{e}\,\mathbf{a}\mathbf{n}\mathbf{t}\mathbf{a}\mathbf{g}\mathbf{o}\mathbf{n}\mathbf{i}\mathbf{s}\mathbf{t}\mathbf{i}\mathbf{c}\,\mathbf{e}\mathbf{f}\mathbf{f}\mathbf{i}\mathbf{c}\mathbf{i}\mathbf{e}\mathbf{n}\mathbf{c}\mathbf{y} = (\text{A} - {\text{A}}_{\text{o}})/ \text{A} \times 100$$

Wherein A denotes the absorbance of the untreated control and A_o_ denotes the absorbance of the treated samples.

#### Anti-microfouling activity

Initially, a sterile glass slide was coated with 200 µg/ml of CuTiNCs and dried at 60 °C followed by washing off with distilled water in order to check fixation and self-cleaning properties, as described by Veena et al. [52]. The coated glass slide was fixed inside a 250-mL Erlenmeyer flask contained 50 mL of seawater supplemented with 50 mL of nutrient broth. The flasks were incubated at 30 °C for 24 h. An untreated control flask was prepared and incubated under exact conditions. At the end of incubation period, an adhered marine biofilm consortia on the surface of the glass slides were washed to remove any non-adherent cells, crushed with sterile scalpel under aseptic conditions, suspended in LB broth and incubated at 30 °C for 24 h to estimate quantitatively the antifouling performance of CuTiNCs [53, 54]. Meanwhile, Light microscope and SEM were utilized to visualize the anti-microfouling effect of CuTiNCs.

### Environmental effluents disinfection

The power of the CuTiNCs in curtailing the microbial load was assessed in two real wastewater samples. One of them was municipal and the other one was an agricultural wastewater sample; their physicochemical criteria were defined formerly according to [55]. About 100 ml of effluents were exposed to two doses (100 and 200 µg/ml) of the as-prepared CuTiNCs, mixed well and incubated for 2 h at room temperature. The total bacteria (TPC), total mold &yeast (TMY), total *coliforms* (TCs) and fecal *Streptococcus* (FS) count were determined using pour plate method on plates of Plate Count Agar (PCA), Rose Bengal chloramphenicol (RBCA), Violet Red Bile Agar (VRBA) and m-Enterococcus agar, respectively, as described by Standard methods for the examination of water and wastewater, 2017 [56]. The plates were incubated at 30 °C for 48 h for bacteria and 25 °C for 72 h for mold & yeast. After incubation period, the colonies were enumerated and expressed as CFU/mL. In addition, the previous parameters in untreated control samples were defined in parallel. The disinfection potentiality was calculated according to the following equation [57].

2 $$\begin{array}{l} \mathbf{D}\mathbf{i}\mathbf{s}\mathbf{i}\mathbf{n}\mathbf{f}\mathbf{e}\mathbf{c}\mathbf{t}\mathbf{i}\mathbf{o}\mathbf{n}\,\mathbf{p}\mathbf{o}\mathbf{t}\mathbf{e}\mathbf{n}\mathbf{t}\mathbf{i}\mathbf{a}\mathbf{l}\mathbf{i}\mathbf{t}\mathbf{y} \left(\mathbf{\%}\right)\\= \text{C}\text{o}\text{l}\text{o}\text{n}\text{y}\,\text{c}\text{o}\text{u}\text{n}\text{t}\,\text{i}\text{n}\,\text{u}\text{n}\text{t}\text{r}\text{e}\text{a}\text{t}\text{e}\text{d}\,\text{s}\text{a}\text{m}\text{p}\text{l}\text{e}\text{s}\\- \text{C}\text{o}\text{l}\text{o}\text{n}\text{y}\,\text{c}\text{o}\text{u}\text{n}\text{t}\,\text{i}\text{n}\,\text{t}\text{r}\text{e}\text{a}\text{t}\text{e}\text{d}\,\text{s}\text{a}\text{m}\text{p}\text{l}\text{e}/ \text{C}\text{o}\text{l}\text{o}\text{n}\text{y}\,\text{c}\text{o}\text{u}\text{n}\text{t}\,\text{i}\text{n}\,\text{u}\text{n}\text{t}\text{r}\text{e}\text{a}\text{t}\text{e}\text{d}\,\text{s}\text{a}\text{m}\text{p}\text{l}\text{e} \text{*}100\end{array}$$

### Statistical analysis

The results of all experiments were expressed as mean ± standard error of mean (SEM); Tukey post-hoc analysis of variance (ANOVA) was utilized to determine the significance of treatments (*p* < 0.05), by Graphpad Instat software [44].

## Results and discussion

**Fig. 1:**
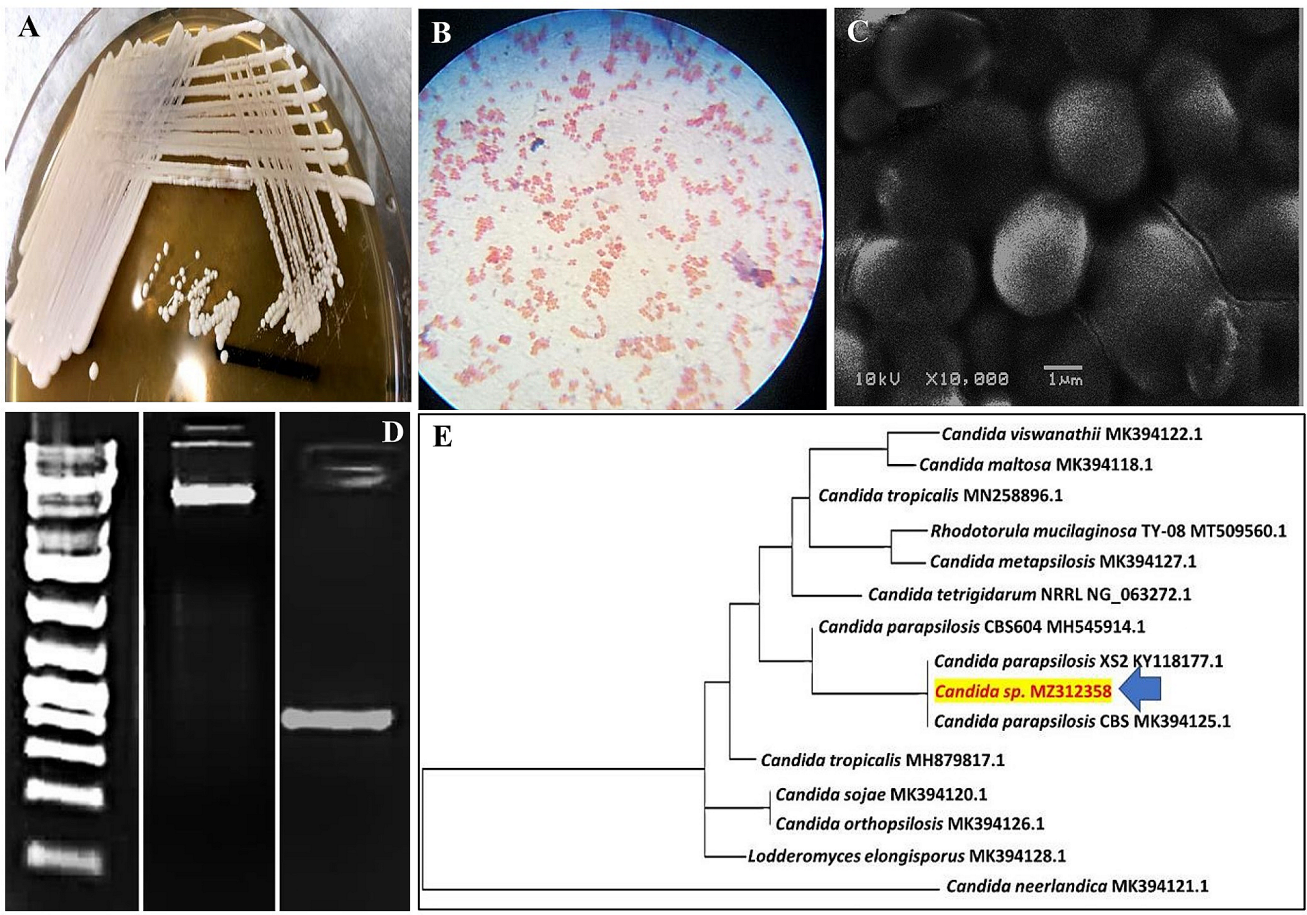
Culture, morphological and molecular characteristics of *Candida sp. MZ312358* (**A**)- Colony appearance on MRS agar, (**B**)-Cells under bright field microscopy (x100), (**C**)- SEM micrograph of cells (X10000) (**D**)- The isolated DNA and ITS- rDNA gene of strain under study and (**E**)- Phylogenetic position of the selected strain by neighbor-joining tree

Initially, the sign of reduction of the NPs precursors and NPs-NCs synthesis could be inferred through visual inspection. Wherein, 7 microbial isolates alternated the media color surrounding the colonies on the agar medium; however, the isolate designated as ASM exhibited the highest tolerance and reduction capability for both NPs precursors (data not shown). Therefore, it was selected as a bionanofactory for synthesis of CuNPs, TiNPs and also CuTiNCs. On MRS agar, their colonies appeared round and moderate in size with a creamy/ whitish appearance and raised borders with characteristic yeast scent. It possesses the ability to grow under aerobic and anaerobic conditions but at a slower rate anaerobically. Besides, it exhibited a considerable capability to grow on broad pH and temperature ranges (4–9, 10 ℃ − 40 ℃) with optimum pH of 6–8 and temperature of 25–30℃, however, the above and below ranges of pH /temperature influenced adversely on its viability. As a unicellular eukaryote, it was a non-motile oval to spherical shape with 4.5 μm width and 5.7 μm length (Fig. 1). In addition, the isolate was identified molecularly by sequencing of ITS- rDNA (≈ 530 bp), which displayed 99% sequence correspondence with all species of the genus *Candida* with lower percentages with other genera. The nucleotide sequence was deposited in GenBank under the accession number of MZ312358. The Phylogenetic relationship of the strain under study and other closely related species was symbolized using the neighbor-joining (NJ) approach. In the phylogram, it was grouped in the main subcluster with two different strains of *Candida parapsilosis*, revealing their phylogenetically close relationship. Broadly, it affiliates to the domain Eukaryota, kingdom Fungi, division Ascomycota, class *Saccharomycetes*, order *Saccharomycetales* and family *Saccharomycetaceae*.

### Mycosynthesis approach and physicochemical characterization of NPs-NCs

**Fig. 2:**
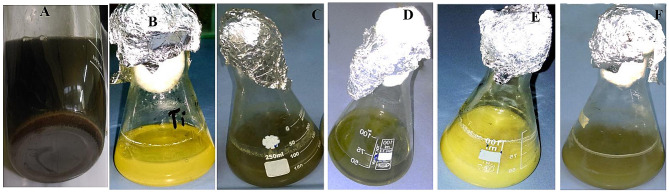
Mycosynthesis of NPs-NCs. **A**, **B** and **C** show the intracellular synthesis of CuNPs, TiNPs and CuTiNCs, respectively. **E**, **F** and **G** indicate the extracellular synthesis of CuNPs, TiNPs and CuTiNCs, respectively

The selected strain *Candida sp.* was subjected to fabricate CuNPs, TiNPs and CuTiNCs both intracellularly and extracellularly. Its ability for the mycosynthesis process was initially assessed by changing the media’s original yellowish-orange colour to brown-olive, whitish and brown-whitish colours corresponding to CuNPs, TiNPs and CuTiNCs, respectively (Fig. 2). Interestingly, the formation of colour is considered a preliminary gauge on the excitation of surface Plasmon vibrations of metal particles that display uniquely different size, crystallinity, and polydispersity properties of metal sols, which are broadly altered at the nanoscale as described by [58]. Comparatively, and in parallel control sets, no particular noticeable variations were found, showing that the biotransformation of metal ions to relevant NPs proceeds mainly in the presence of the possible reducing agents provided by the bionanofactory *Candida sp.* The characteristic traits and structural identification of mycologically prepared NPs-NCs were elaborately evidenced via a number of analytical techniques as would be displayed.

### Morphological properties

**Fig. 3:**
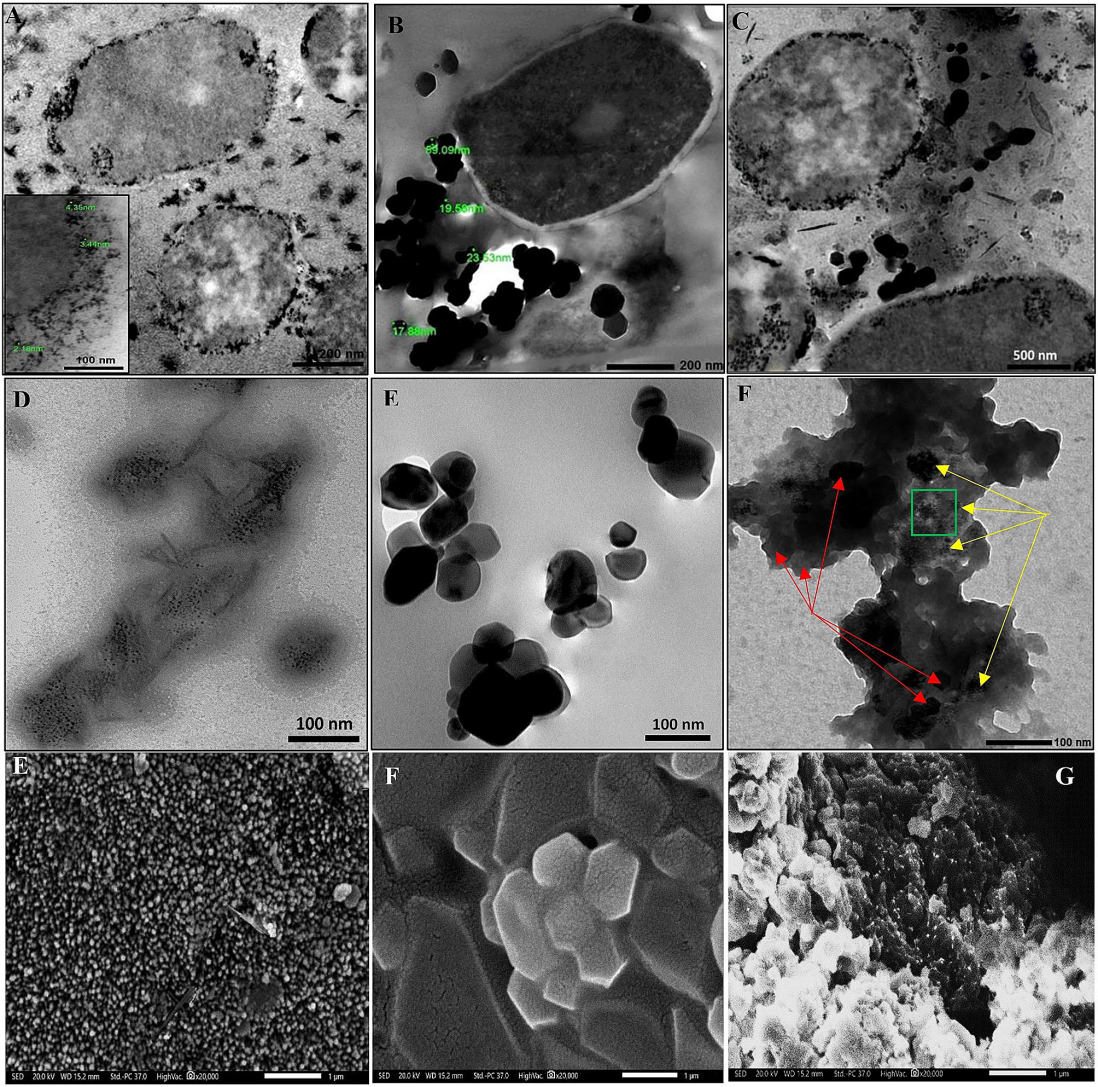
Electron microscopy examination of CuNPs, TiNPs and CuTiNCs fabricated by *Candida sp.* (**A, B** and **C**) TEM micrographs showing intracellular CuNPs, TiNPs and CuTiNCs, respectively. (**D, E** and **F**) TEM micrographs of extracellularly- synthesized CuNPs, TiNPs and CuTiNCs, respectively. Red arrows referred to TiNPs and yellow arrows referred to CuNPs. The squared area was examined by EDX. (**E, F** and **G**) SEM micrographs of extracellularly- synthesized CuNPs, TiNPs and CuTiNCs, respectively

To ascertain the capability of *Candida sp.* in NPs-NCs fabrication, either extracellularly or intracellularly, electron microscopy (transmission (TEM) and scanning (SEM)) was employed. It gives an insinuation about morphology, dimensions, textural properties, location and interior structure of nanomaterials [59]. Obviously as depicted in (Fig. 3), there was heterogeneity in the morphologies, size and localization of NPs-NCs. The cells of *Candida sp.* possessed the ability to synthesize CuNPs both intracellularly and extracellularly. They seemed as numerous, uniform, electron opaque, tiny and spherical or quasi-spherical nanoparticles ranging in their size from 2.18 to 24.85 nm in a monodispersed pattern or with slight aggregation scattered at the periplasmic compartment of the cell. Besides, rod-shaped CuNPs dispersed among the cells were also visualized. Meanwhile, our fungal cells failed to internalize and reduce the parent molecule of Ti inside the cells to its NPs counterpart but succeeded in this mission extracellularly. It is worth mentioning that such synthesis process was implemented via extracellular fungal metabolites such as enzymes, terpenoids, extracellular polysaccharides, polyketides and non-ribosomal peptides. The mycosynthesized TiNPs varied in their shape from spherical, rods to polygonal and size oscillated from 17.8 to 98.3 nm appeared as clusters in aggregates surrounding the cell. However, the nanofactory *Candida sp.* exhibited a prominent capability in simultaneous synthesis of both CuNPs and TiNPs in nanocomposites form upon exposing to the presursore salts of both metals.

On the other hand, the cell free supernatent of fungal culture shared the same features with the producing cells in the overall NPs morphology and size. Where, TEM and SEM micrographs illustrated that CuNPs were dark, tiny, spherical or needle-shape embeded in less dense light funagl protenaceous matrix. The presence of rod or needle shaped CuNPs could be attributed to Ostwald ripening mechanism [60]. While, larger irregular, square, rectangular and polygonal TiNPs were marginally agglomerated. Regarding CuTiNCs, they displayed as an undefined assemblage of polygonal TiNPs with well-defined edges that were agglomerated arbitrarily into the matrix of fungal biomolecules, which appeared as electron opaque bulk or spots with sizes ranging from 15 to 75 nm. Intriguingly, CuNPs ornamented the surface of TiNPs as indicated by arrows (Fig. 3). Such agglomerated NCs were reported frequently in metal hybrid nanostructures [44, 61, 62]. It could be assigned to the electrostatic attractions between NPs, their surface energy differences, the magnetic interference and the coexistence of H-bonding in the microbial bioactive moieties accompanying to the NPs as well [50, 63, 64]. Broadly, the variations in NPs-NCs morphology and size was documented previously in litratures by [44] and [65], who attributed that to the selective interaction mechanism, microbial biochemistry, metal type and the entire physical conditions of the synthesis reaction.

Virtually, the features of NPs-NCs seemed to be equivalent for both extracellular and intracellular synthesis approaches. Therefore, the extracellularly produced NPs-NCs were selected for further characterization and application steps. It symbolizes easy implementation, higher yields, saving more time/effort and cost-effectiveness by the dint of lacking extra extraction and purification steps, compared to intracellular approaches.

### Optical properties

**Fig. 4:**
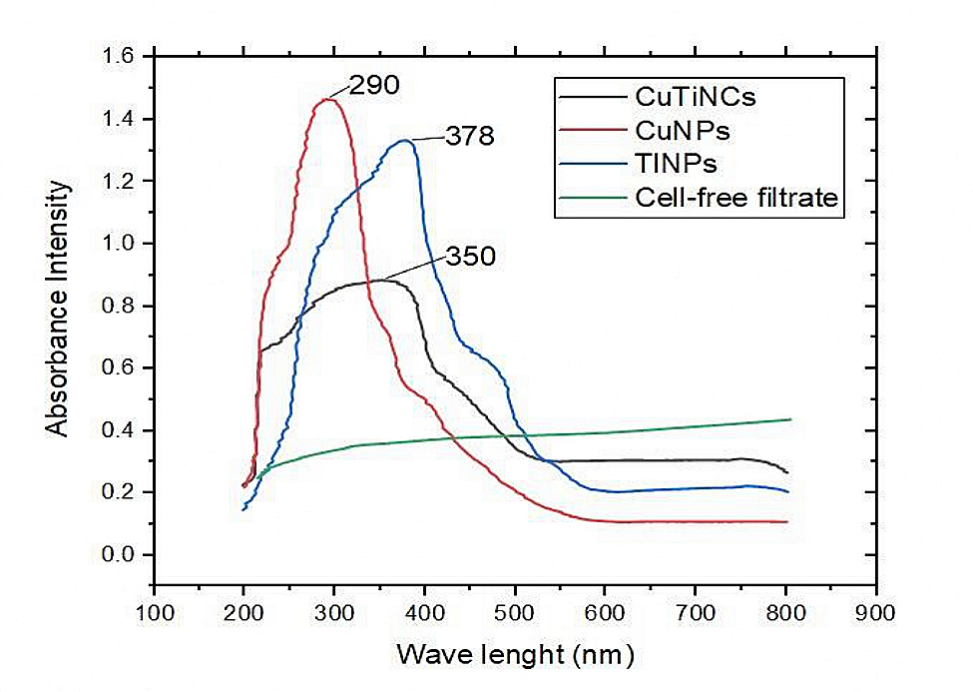
UV-spectrophotometric analysis of CuNPs, TiNPs and CuTiNCs fabricated by *Candida sp.*

Typically, UV-Vis spectroscopy is a low-cost, quick, and non-destructive initial instrumental technique for the recognition of NP systems, and types besides monitoring the signature of colloidal particles in the range of 200–900 nm [47, 66]. As marked out in (Fig. 4), each mycosynthesized metallic sole NPs exhibits distinct absorbance bands in distinctive representative spectra. A relatively narrow, well-defined surface Plasmon resonance (SPR) band with high absorbance was observed at wavelengths of 290 and 378 nm for copper and titanium, respectively. This finding is consistent with previous observations in which the UV-vis spectra for CuO NPs ranged between 250 and 300 nm [67–69]. Meanwhile, some studies presented a spectrum in which the absorption peaks of around 380 and 400 nm correspond to TiNPs anatase phase, with the cutoff wavelength at 379 nm [70, 71]. Notably [45], found that SPR of bacterially synthesized TiNPs at 360 nm. whereas [72], detected it at 380 nm, which were synthesized by *Bacillus mycoids*. Generally, the maximum absorption peak related to TiNPs was observed in the range of 300–400 nm [45].

On the other hand, a broader cumulative peak with increased blue-shifted absorption at 350 nm was detected in the combined hybrid system of CuTiNCs. Interestingly, the hybridization of both metal precursors in the reaction mixture of the mycosynthesis system caused a relative change in the absorbance maxima, which appeared at an intermediate place between the SPR band of CuNPs and TiNPs, denoting the formation of hybrid CuTiNCs.

As referred previously by [73], UV–Vis spectroscopy could easily give insinuation about the signal architecture of any binary or hybrid nanomaterials, wherein, the presence of single SPR peak at an intermediate location between the SPR of the two metals indicates a randomly organized hybrid metal/metal oxides. In this sense [36], found that the phyto- synthesized CuO-TiO_2_-NCs displayed their characteristic absorption band at 301 nm; however [74] and [75], detected an absorption peak of chemically synthesized CuO‐TiO_2_-NCs at 380 and 363 nm, respectively,. Such variations in results could be attributed to the differences in particle surface properties (e.g. particle morphology, crystallinity, size and aggregation state, etc.) owing to synthesis method, precursor type, precursors concentration, reducing agent type/concentration, capping agent type/concentration and overall solution chemistry [68, 76–78]. In addition, the presence of a single surface Plasmon band for each NPs-NCs with a tailing appearance might reveal the formation of small aggregated nanocrystals [79]. Generally, the incorporation of Cu and Ti into CuTi hybrids is more favorable in terms of charge transfer as well as functional photocatalytic activity in the visible range; hence, boosting photoelectrochemical properties throughout a broader spectrum of wavelengths [80].

### Structural properties

**Fig. 5:**
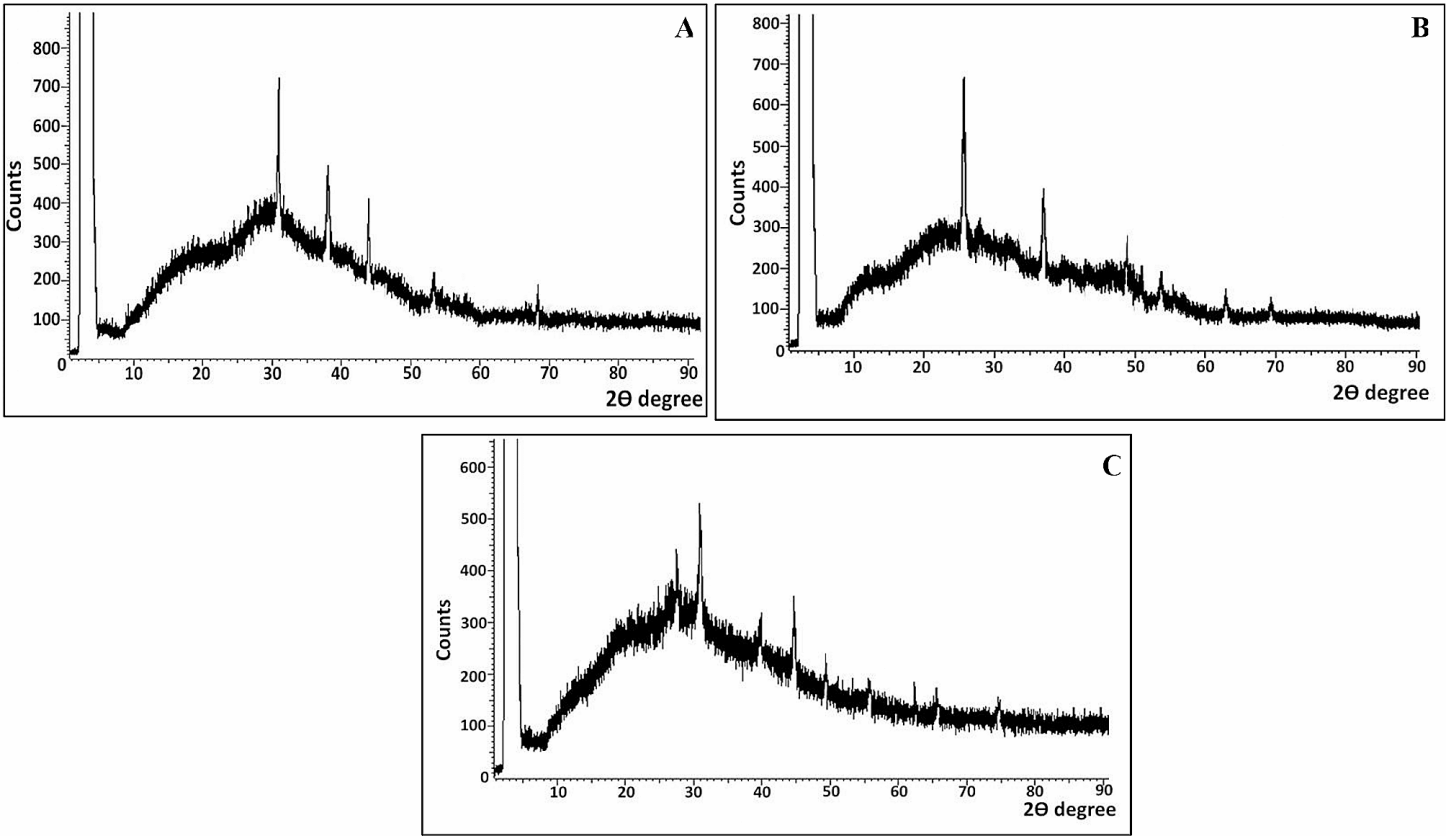
XRD diffractograms of CuNPs (**A**), TiNPs (**B**) and CuTiNCs (**C**) fabricated by *Candida sp.*

The crystalline nature, phase purity and identity of mycosynthesized CuNPs, TiNPs and CuTiNCs were scrutinized and confirmed via XRD analysis (Fig. 5). As observed, a series of characteristic peaks in the XRD pattern of the examined CuNPs at 2*θ* = 30.9°, 39.39°, 44.91°, 53.57°, and 68.88°, which correspond to (110), (111), (− 202), (020), and (200), Bragg’s reflection, respectively, matched the diffraction planes of monoclinic cubic phase of CuO (JCPDS file no. 00-005-0661) [81]. However, the crystalline structure of the TiNPs exhibited predominant mycosynthesis of crystalline anatase with characteristic peak positions at 25.8°, 37.9°, 48.7°, 54.8°, 62.9° and 70.7°, which matched the diffraction planes of (101), (004), (200), (105), (211) and (204), respectively, based on (JCPDS file no. 2-21-1272). These findings were compatible with previous reports of greenly synthesized CuO and TiO_2_-NPs [45, 81]. Notably, the presence of CuO and TiO_2_ distinctive peaks in the XRD diffractogram of CuTiNCs ascertained the successful fabrication of hybrid composites. Wherein, clear diffraction peaks of CuO at 30.9°, 39.4°, 44.91 indicated the presence of CuO in the nanocomposites along with diffraction peaks of TiO_2_ at 25.8°, 48.9°, 54.8°, 62.9° and 75.1°. Obviously, there was no shift in the diffraction peaks of TiO_2_ displayed in the CuTiNCs upon incorporation of CuO, proposing the adherence of CuNPs on the surface of TiNPs but not integrated into the lattice of TiO_2_. This suggestion was harmonized formerly with TEM. Our result is in compliance with that reported by [82] who also declared that the absence of notable changes in the intensity and half-height widths of TiO_2_ diffraction peaks revealed the impregnation of CuO without changing both structures or even the crystallite size of TiO_2_ in the chemically synthesized xCuO/TiO_2_ nanocomposites. On the other hand, in the study performed by [83], only sharp peaks concerning Cu were displayed in an amorphous TiO_2_ matrix of Cu@TiO_2_ samples, which were prepared by sole gel method; reflecting the advantageous proper incorporation of both CuO and TiO_2_NPs in our hybrid crystalline system. As a general notice, all XRD patterns of our examined NPs-NCs showed a background hump at 2θ before 20°, implying the conjugation of fungal biomolecules with the crystalline as-synthesized NPs-NCs. Likewise, a similar finding was recorded by biologically synthesized NPs [84, 85].

### Compositional properties

The elemental composition and quantification of the NPs samples were defined through energy-dispersive X-ray analysis (EDX). The elemental pattern of CuNPs indicated characteristic major absorption peaks at 0.9 and 8 keV with an atomic percentage of 39.6%, which corresponds bending energy of metallic copper ; also an additional peak at 0.5 keV that signifies oxygen with an atomic percentage of 7.3%. Regarding TiNPs, a typical maximum peak at bending energies of Ti and O at 4.5, 4.9 and 0.5 keV with atomic percentages of 41.1% and 50.4%, respectively (Fig. 5). As implied from the obtained previous EDX profiles, the examined CuNPs and TiNPs were biosynthesized in their oxide forms. Remarkably, our finding is congruent with that obtained by [44, 45, 86]. On the other hand, the binary hybrid structure of CuTiNCs was emphasized as indicated by the predominance of Cu, Ti and O signals in the examined samples in atomic percentages recording 31.7, 35.3 and 25.5%, respectively. Notably, the slightly higher percentage of Ti signal than Cu implied the uniform dispersion of CuO on the surface of TiO_2_NPs as pointed out by [44] and [82], which agreed our finding that also recorded formerly by TEM and XRD analysis. In addition, obvious intense peaks of N and S were detected at 0.39 and 2.3 keV in CuTiNCs pattern with atomic percentages reached to 1.7 and 2.1, respectively. The presence of such elements could be ascribed to the conjugation of fungal biomolecules (e.g., proteins, lipids, etc.) with the biosynthesized NCs [87, 88]. Besides, the coexistence of strong C signal in all examined EDX profiles of NPs-NCs at 0.27 KeV was considered residues from fungal carbonaceous metabolites. Interestingly, the association of such fungal entities with as-prepared NPs-NCs is an advantageous trait through furnishing them with functionality, stability and dispersity as self-functionalizing capping agents; hereby, avoid multiple surface modification steps as mentioned in chemical and physical methods. The involvement of microbial biomolecules with NPs-NCs was documented frequently in the green synthesis approach [89]. However, despite the significance of such biomolecules as functionalizing agents, especially in biological applications (e.g. anticancer, antioxidant, antimicrobial, etc.) [90], employed 500 °C calcination to TiNPs for eradicating the organic scaffolds conjugated with NPs derived from gum matrix to get refined free of companion biomolecules.

### Functional properties

The analysis of FTIR spectroscopy is a powerful tool for the primal determination and detection of active microbial biofunctional groups associated with the synthesized NPs, via absorbing the corresponding “resonant frequencies” and depicting their representative IR spectra in the infrared range of (4000 –400 cm^− 1^) [70]. FTIR spectra of the mycosynthesized CuNPs, TiNPs, CuTiNCs and cell-free supernatant are illustrated in (Table 1; Fig. 7); reflecting the presence of common peaks that contributed substantially in their biosynthesis, stabilization and functionalization.

**Table 1:** FTIR peaks assignments and their corresponding functional groups associated with CuNPs, TiNPs, CuTiNCs and cell-free extract involved in their biosynthesis and capping

Wave number (cm^− 1^)	Vibration type	Assignment	Reference
3700–4000	stretching	O–H group	[141]
3440, 3441 and 3442	stretching	NH_2_	[142]
2936	stretching	C–H	[143]
2438	stretching	C = O	[144]
2270, 2352 and 2382	stretching	NH^2+^ and NH^3+^ in protein/peptide bonds	[145]
1600, 1611 and 1649	stretching	Amide-I/amide-II linkages	[146]
1421	stretching	C-O	[138]
1447	stretching	–C–H / –CH3 in aliphatic –compounds	[147]
1497	stretching	C = O of carboxylic acid	[146]
1372	stretching	carboxyl groups (–COOH)	[148]
1045, 1076, 1124, 1126 and 1177	stretching	PO_4_^3−^	[149]

**Fig. 6:**
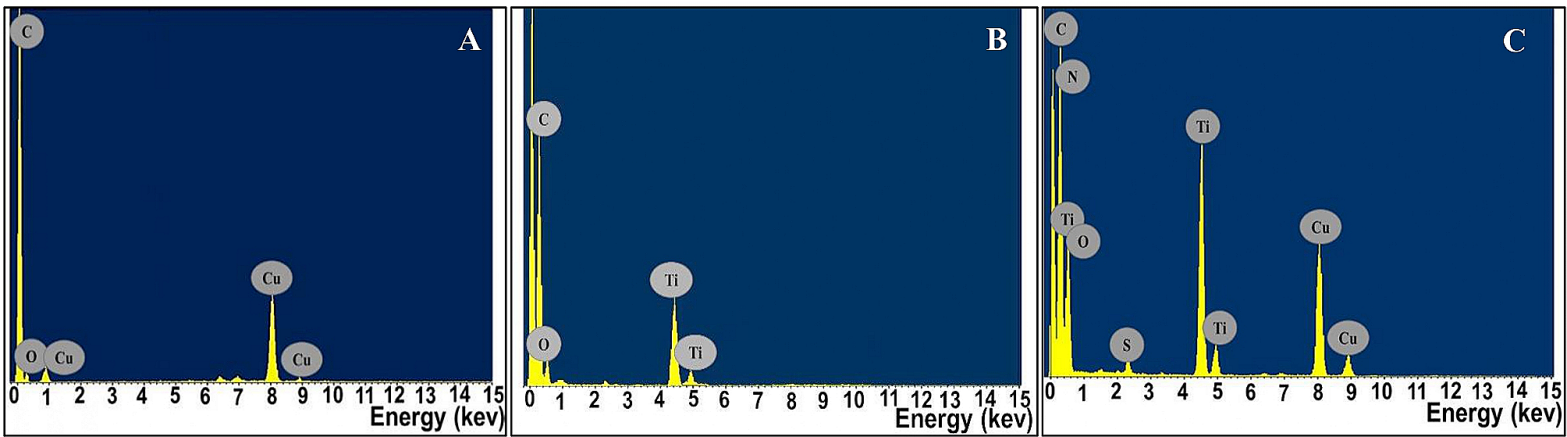
EDX profiles of CuNPs, TiNPs and CuTiNCs fabricated by *Candida sp.*

**Fig. 7:**
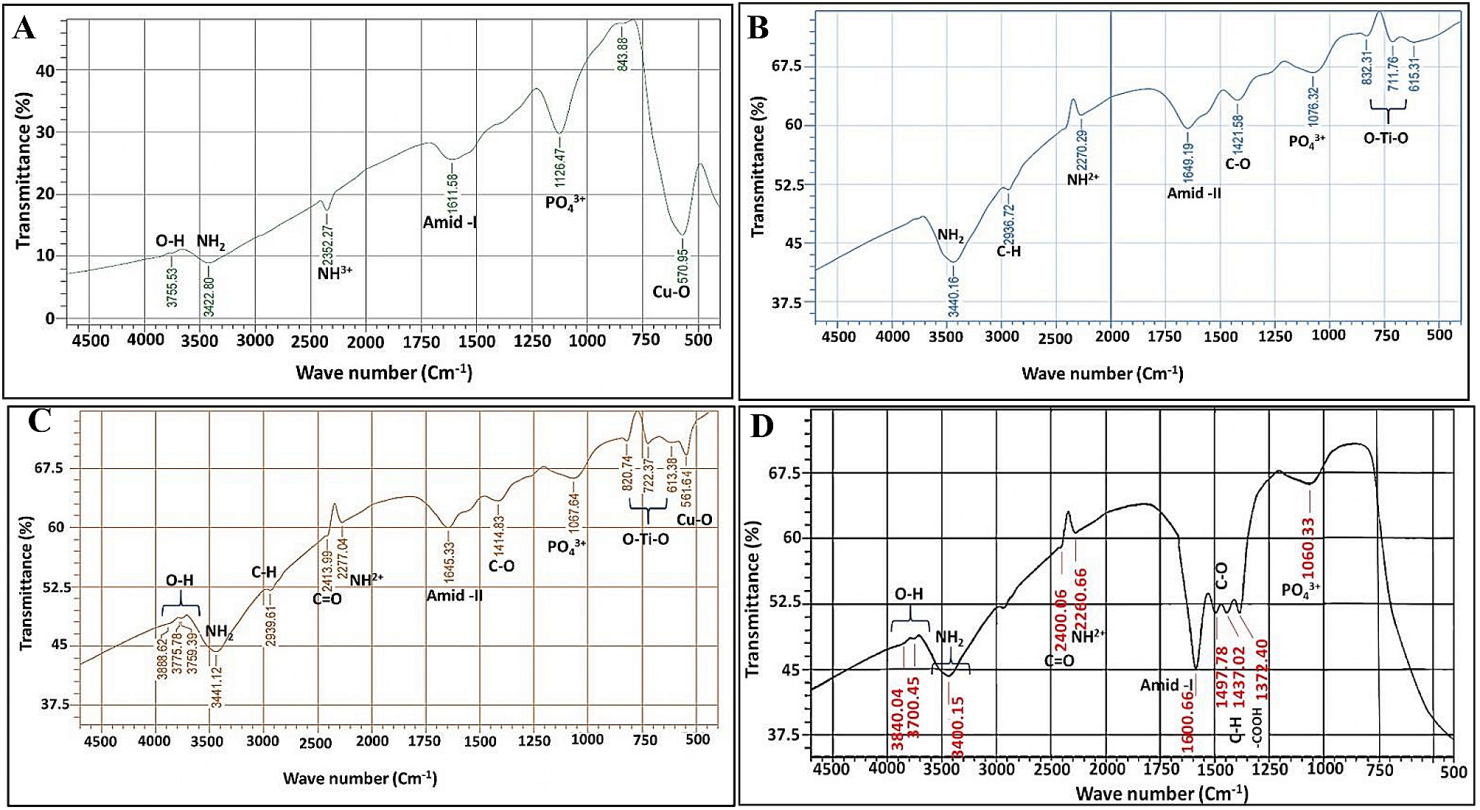
FTIR spectrum of mycosynthesized CuNPs (**A**), TiNPs (**B**), CuTiNCs (**C**) and Cell-free supernatant of *Candida sp.* (**D**)

Generally, our results agreed with [91] and [92] who found the same vibration peak in INPs and AgNPs synthesized by *Pleurotus sp.* and nematophagous fungus *Duddingtonia flagrans*. Concerning the vibrational modes for Cu and Ti [93], manifested that the region below 1000 cm^− 1^ is characterized by metal-oxygen bonds. Hereby, in the fingerprint zone, especially at 570 cm^− 1^ and the range of 615–830 cm^− 1^; respectively, the spectral peaks are affiliated to Cu-O, and O-Ti-O. Such stretching vibrations were aligned with earlier reports of [63] and [94]. However, a minor shift in the absorption peaks was noticed in CuTiNCs profile, compared to both sole metal NPs, which might be traced back to the interaction of CuNPs and TiNPs and the development of CuTiNCs [95, 96]. Sensibly, the coupling of multiple functional groups such as C = O, C-H, C–O–C, PO_4_^3^-, amine and amide with NPs seemed to be propitious. Where, the electrostatic attraction or interactions at the oxygen side form a bridge between metal atoms and free amine groups, exopolysaccharides, protein residues and phospholipids mediated stabilization functionality as dispersing ligands. Similarly [97], advocated the same point of view.

### Thermal properties

**Fig. 8:**
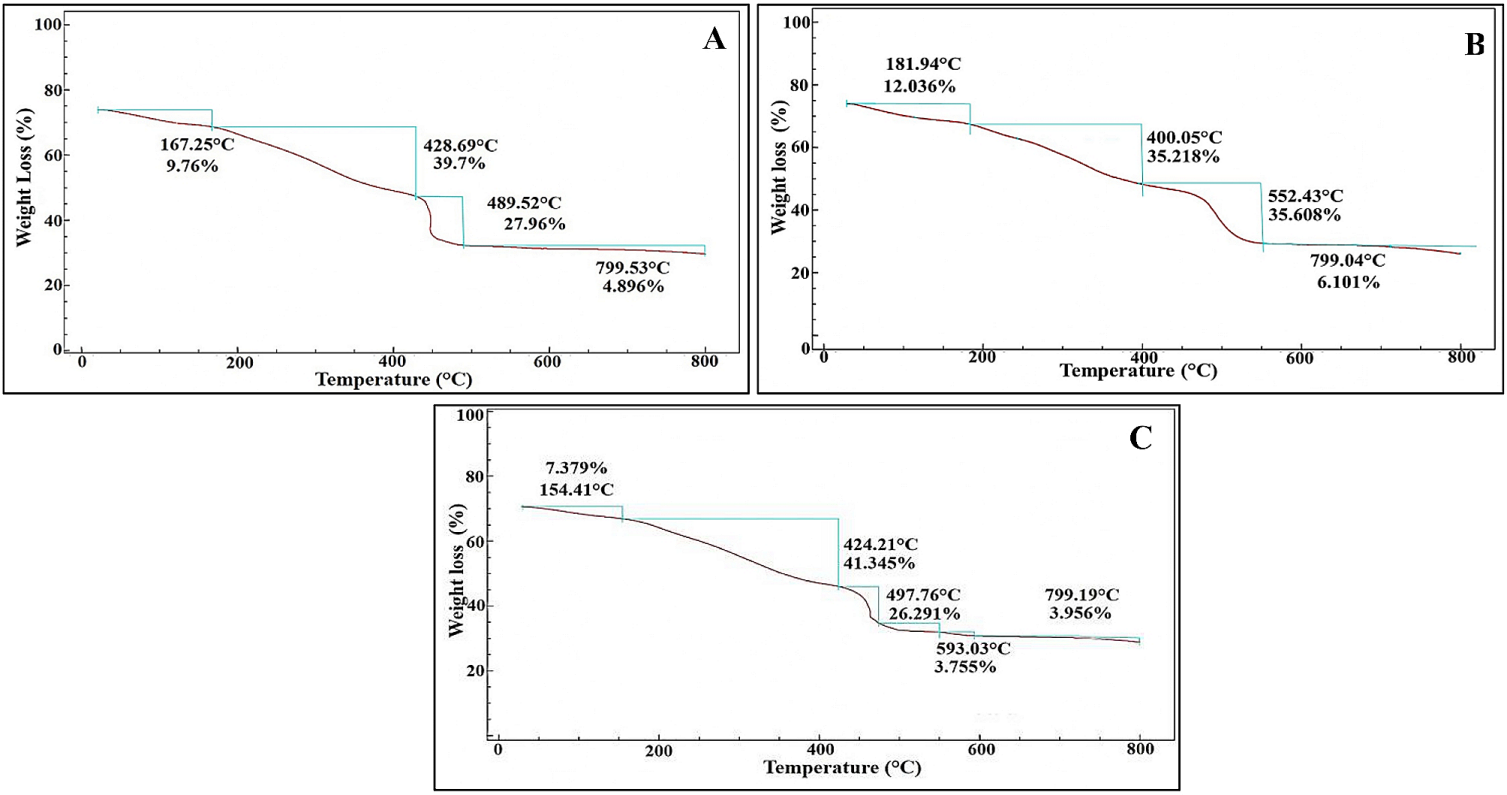
Thermal behavior analysis by TGA for CuNPs (**A**), TiNPs (**B**) and CuTiNCs (**C**) fabricated by *Candida sp.*

The thermogravimetric analysis of the pure biofabricated CuNPs, TiNPs and their nanocomposites retrieved curves were demonstrated in (Fig. 8). Its main idea lies behind following up the correlation between the applied higher temperature in constant rate and mass shift proportions; offering thereby a valuable insight into the mass, stability, thermal properties, coating composition and ratios of the nanoparticles [47]. In temperatures ranging from 20 °C to 800 °C, the TGA curves of the examined NPs-NCs samples were represented in four main weight loss stages. Initially, rapid mass reduction (7.37-12.0%) can be easily detected in a temperature lower than 200 °C corresponding to the desorption and release of chemisorbed and physisorbed/retained water molecules [98]. The second weight loss portion was recorded from 200 to the range of 400–428 °C reached to 39.7, 35.21 and 41.34% for CuNPs, TiNPs and CuTiNCs, respectively, which could be ascribed to the thermal pyrolysis and carbonization of organic molecules bound to the surface of NPs-NCs [98]. The third stage began from 428 °C to the range of 490–552 °C with weight loss assessed by 27.96, 35.60 and 26.29% % for CuNPs, TiNPs and CuTiNCs, respectively. However, an additional step was observed in the case of CuTiNCs, wherein about 3.75% weight reduction was observed at 593 °C, which was attributed to the continuous evaporation of biosorbent water molecule and gradual pyrolysis of the biomolecules residues (e.g., as polysaccharides, proteins, phospholipids, etc.), which still tightly bound to NCs surface [90]. In the same context [99], revealed that the conjugation of amide I signature with inorganic particles that were confirmed through FTIR analysis at wave numbers of 1600: 1650 cm^− 1^, resulted in weight loss at a temperature range of 250–595 °C, which are harmonized with the current data.

Remarkably, as stated by [100], the weight loss at the temperature range of 200–350 °C and 350–550 °C assigned to the degradation of organic matrix followed by the combustion of organic residues. Actually, non-significant weight loss reaching 4.89, 6.1 and 3.95% was observed for CuNPs, TiNPs and CuTiNCs, respectively throughout the range of 490/552°C to 799 °C; implying the stability of materials mass and their crystallinity. Likewise [101], declared that insignificant weight loss in the temperature range of 540∼900℃ was considered a sign of the transformation of TiO_2_ from the amorphous phase to a crystalline phase. Ultimately, about 82.31, 88.96 and 82.73% of examined CuNPs, TiNPs and CuTiNCs, respectively, get destroyed, leaving 17.69% (0.94 mg), 11.04% (0.59 mg) and 17.27% (0.87 mg) with considerable thermal stability remaining. Generally, the hybridization of both Cu and Ti in the composite nanoform didn’t influence adversely on their thermal stability. Conversely [98], found that the pure TiO_2_ offered higher thermal stability with 23% mass loss compared to the Cu3%-TiO_2_ nanostructure that lost 35% of its mass after Cu incorporation.

### Surface charge properties

**Fig. 9:**
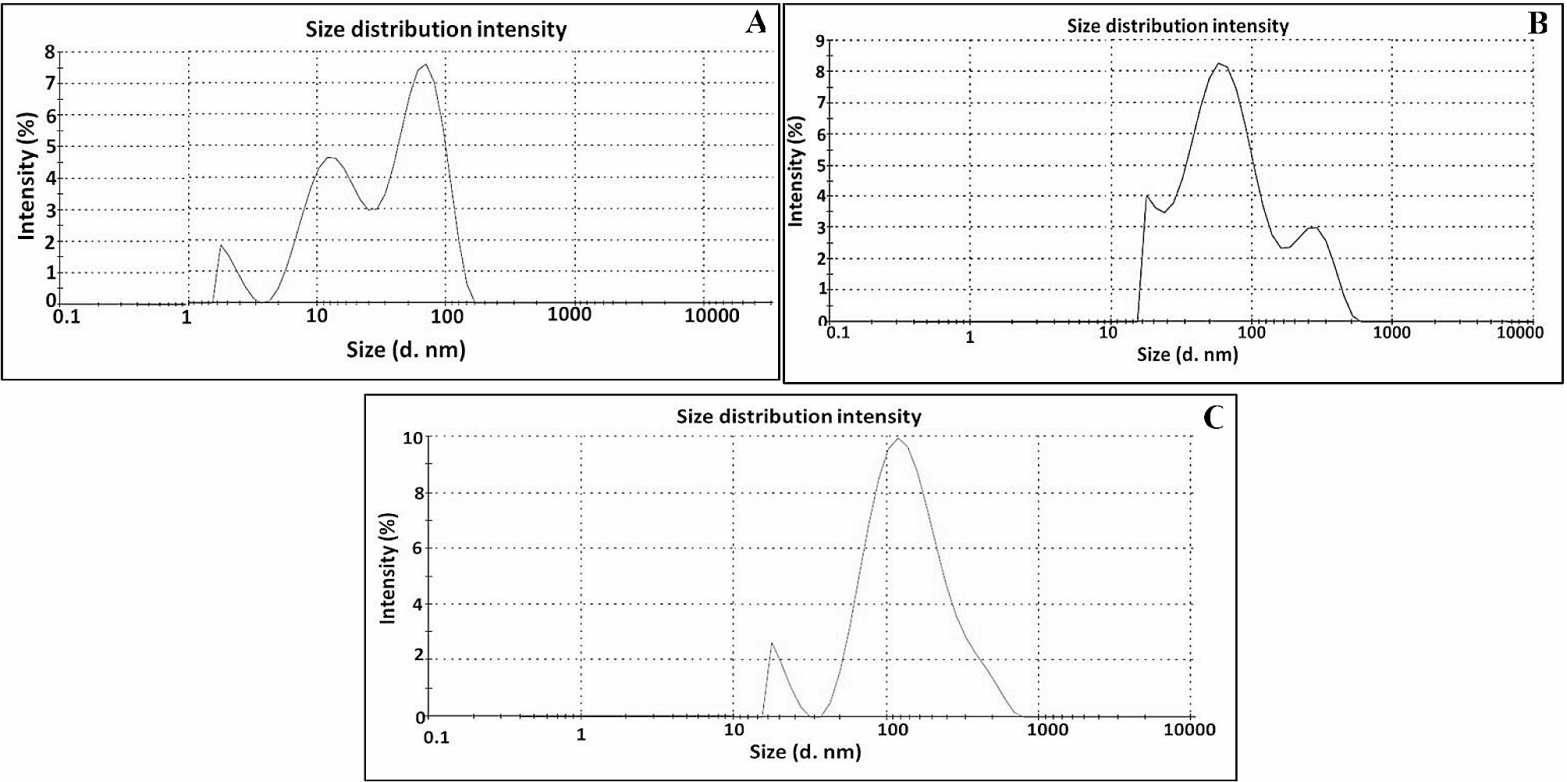
Hydrodynamic size of CuNPs (**A**), TiNPs (**B**) and CuTiNCs (**C**) prepared by *Candida sp.*

To evaluate the manner of particle diffusion in any fluid (i.e., hydrodynamic diameters (size distribution) and *ζ*-potential), the dynamic light scattering (DLS) was employed as a noninvasive technique. As revealed by [88], this technique gives an insinuation about the fluctuations in light scattering intensity as a function of particle sizes, which is attributed to Brownian motion. Figure 9 demonstrated the particle size distribution curves of CuNPs, TiNPs and CuTiNCs. As observed, the hydrodynamic sizes of CuNPs assessed by 84.2 nm (78.9%), 27.3 nm (16.7%) and 3.5 nm (4.4%). However, the hydrodynamic size of 28.8, 83.4 and 393 nm with intensities 14.4, 72.1, and 13.5% was recorded for TiNPs. Regarding CuTiNCs, the obtained hydrodynamic size were 28.8 nm (24.3%) and 171.5 (75.7%) nm. Such distinct difference in size between DLS and TEM could be ascribed to the conjugation of water and other fungal moieties to the surface of NPs-NCs [88].

**Fig. 10:**
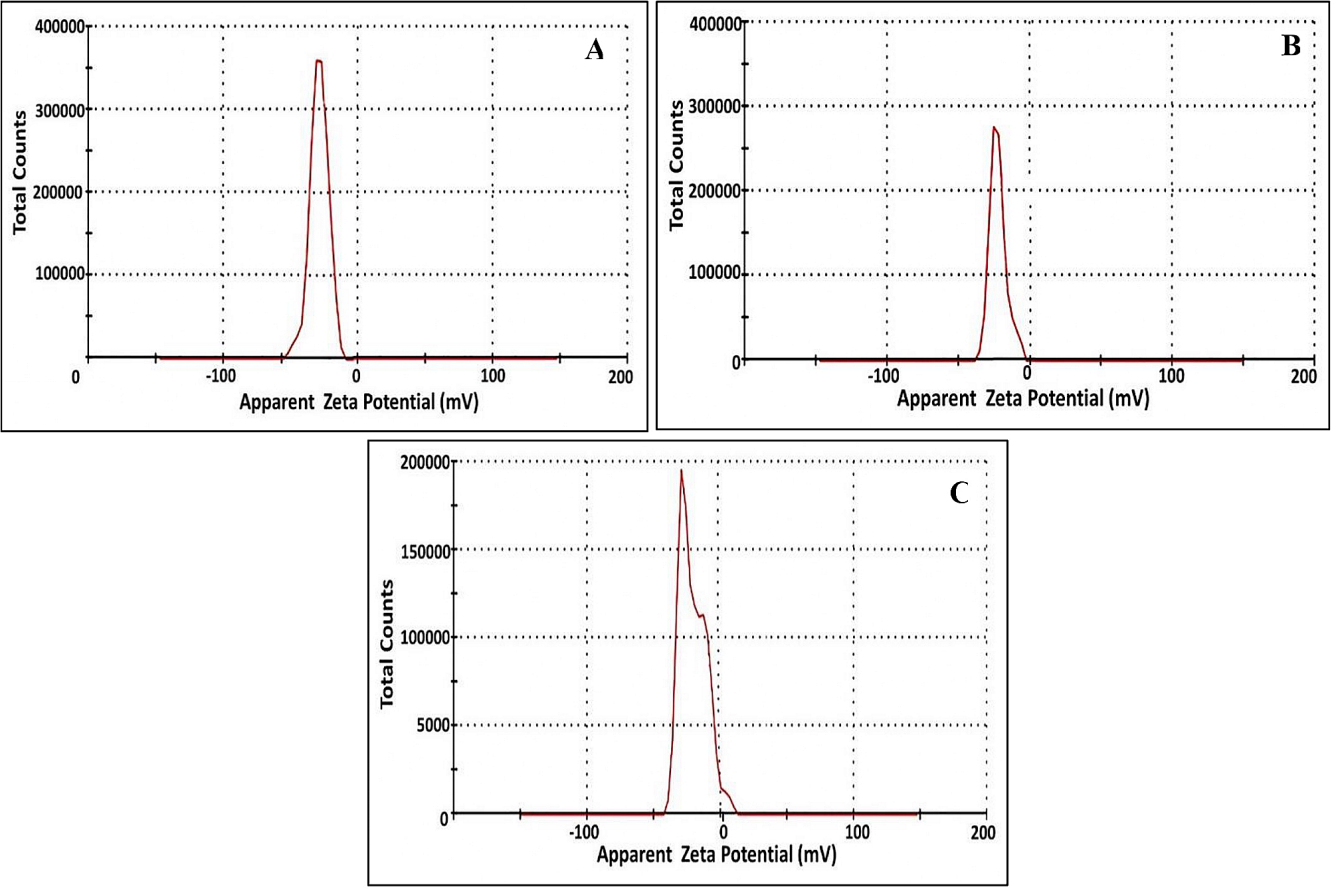
Long-term stability analysis determined by Zeta potential for CuNPs (**A**), TiNPs (**B**) and CuTiNCs (**C**) synthesized by *Candida sp.*

On the other hand, the storage stability of NPs colloidal dispersion, surface charge, degree of hydrophobicity, particles distribution as well as the manner/ fate of interaction with the biological systems are predicted by measuring the zeta (ζ) potential [46, 102]. This is determined by the magnitude of the attractive or repulsive forces, which in turn is correlated to the potential difference between the outer Helmholtz plane and the surface of shear [46]. Accordingly, the stability of the nanosuspension requires the value of zeta potential to be in the range of ± 20–30 mV [33, 47]. The higher absolute zeta potential of NPs indicates the high electric charge on their surfaces, which points to strong repelling forces that stabilize the NPs in the medium with minor agglomeration [47]. As demonstrated in the zeta graphs (Fig. 10), the electrokinetic that existed on the shear plane of a particle, which is related to surface charge, were evaluated by -31.3, -26.2 and − 27.7 mV for CuNPs, TiNPs and CuTiNCs, correspondingly. As displayed, CuNPs possessed significantly greater zeta potential owing to electrostatic repulsion force among the NPs, which exceeded Van der Waals attraction force that in turn results in “Brownian motion”, ultimately higher stability and lower propensity to agglomeration [102, 103]. In general, the electrostatic repulsion force among similar surface-charged particles is explained by DLVO theory [103]. On the other hand, the lower ζ potential value of TiNPs, compared to the other examined NPs-NCs, could be simply assigned to the larger size and non-uniform shape of TiNPs, which generated heterogenous dispersity and slow particle diffusion in light scattering intensity as reflected by [104]. Notably, all examined NPs-NCs were negatively charged owing to their wrapping with anionic biomolecules of negatively charged proteins, nucleic acids, lipopolysaccharides, etc., which bestowed NPs-NCs with dispersity, functionality and stability. Seemingly, all the previous data are harmonized, complementary and confirming each other. Interestingly, the data of as-prepared NPs-NCs of the current study showed superior ζ-potential than other related studies [105, 106].

Thence, it is plausible to propose the mycosynthesis/functionalization mechanism and also highlight the eminant performance of nanofactory *Candida sp.* in its manipulation differently with both NP-parent molecules, based on their nutritional importance to the cells or their toxicity. That was unequivocally evident via cellular compartmentaization of each synthesized NPs and even NCs and also in exhibiting variable dimensions and overall properties [42, 107]. For the intracellular synthesis of CuNPs, the intact cells internalized Cu-precursor through electrostatic interaction between positively charged Cu^2+^ ions and negatively charged functional groups that scattered on the intact cell; followed by metabolizing them either to alleviate their toxicity or to recruit them in their metabolic functions [94]. In this context, it is worth stating the significant tasks of Cu as one of the essential microelements, which contributes in physiological catalysis, regulation of the electron transport process, denitrification, oxidative respiration and other redox reactions [44]. After Cu^2+^ ions uptake, the yeast cells underwent a cascade of oxidation-reduction steps via reducing NADH-dependent enzymes (e.g., hydrogenases, cytochrome reductase, nitrate reductase, superoxide dismutase dehydrogenases, and catalase). Such reducing biomolecules shuttle electrons to the metal ions, which were subjected to sequential redox reaction led to the formation of CuO nuclei. With continuous nucleation, the growth of larger crystals and self-assembly were also continued till reaching to the most thermostable crystallographically oriented form, as explained in detail by [108].

At this stage the *Candida sp.* cells accumulated CuNPs in the periplasmic compartment either for storage or for pumping out by the efflux system to maintain homeostasis [44, 78, 104]. However, the toxicity of the Ti-parent molecule could explain its transformation extracellularly by metal-selective interaction strategy induced by *Candida sp.* cells, which adopted TiNPs synthesis under the umbrella of metal adaptation/detoxification scenario. The cells reduced Ti ions outside the cells, rather than internalize them, via several extracellular reducing biomolecules such as extracellular polysaccharides, enzymes, etc [109]. It is proposed that TiNPs were synthesized via serious collaboration of such biomolecules to nucleate Ti ions in the form of TiO_2_-nuclei, which undergo a growth stage to form pleomorphic TiNPs. However, upon dual exposure to Cu and Ti precursors, the cells are subjected to lethal metal stress, which triggers the cells harness multiple metabolic pathways simultaneously for detoxifying both metal ions in the form of CuTiNCs. Seemingly, all biosynthesized NPs-NCs were less toxic than their parent molecule. That could be clearly evident through TEM, which depicted the viability and healthy appearance of the cell. The cells were still intact with no evidence of cellular damage or wall rupturing; reflecting the capability of our bionanofactory to exert a balance for healthy growth with efficient metal detoxification.

On the other hand, the availability of reducing biomolecules in the free-cells supernatant (i.e., extracellular mycosynthesis) such as enzymes, quinones-derivatives, quorum sensing molecules (e.g., farnesoic acid, tyrosol, farnesol, tryptophol, and phenylethyl alcohol), extracellular polysaccharides and other secondary metabolites [109] facilitated the transformation of both metal salts into their corresponding metal oxides in a sequential stages of oxidation-reduction reactions as proposed in the possible following equations:

3 $${\text{C}}_{6}{\text{H}}_{12}{\text{O}}_{6} \to 2{\text{C}}_{2}{\text{H}}_{5}\text{O}\text{H} + 2{\text{C}\text{O}}_{2} + \text{O}\text{x}\text{i}\text{d}\text{o}\text{r}\text{e}\text{d}\text{u}\text{c}\text{t}\text{a}\text{s}\text{e}$$

4 $${\text{H}}_{2}\text{O}\to {\text{H}}^{+}. + \text{O}\text{H}$$

5 $${\text{C}\text{u}}^{2+} + {\text{N}\text{A}\text{D}\text{H}}^{+} \text{H}+ + \text{N}\text{A}\text{D}\left(\text{P}\right)\text{H}-\text{R}\text{e}\text{d}\text{u}\text{c}\text{t}\text{a}\text{s}\text{e} \to 2{\text{C}\text{u}}^{+} + {\text{N}\text{A}\text{D}}^{+} + 2{\text{H}}^{+}$$

6 $${\text{T}\text{i}}^{4}+ + \text{N}\text{A}\text{D}\text{H} + {\text{H}}^{+} + \text{N}\text{A}\text{D}\left(\text{P}\right)\text{H}-\text{R}\text{e}\text{d}\text{u}\text{c}\text{t}\text{a}\text{s}\text{e} \to {\text{T}\text{i}}^{3}+ + {\text{N}\text{A}\text{D}}^{+} + 2{\text{H}}^{+}$$

7 $${\text{T}\text{i}}^{3+} + \text{N}\text{A}\text{D}\text{H} + 3{\text{H}}^{+}. +\text{N}\text{A}\text{D}\left(\text{P}\right)\text{H}-\text{r}\text{e}\text{d}\text{u}\text{c}\text{t}\text{a}\text{s}\text{e} \to {\text{T}\text{i}}^{2+} + {\text{N}\text{A}\text{D}}^{+} + 3{\text{H}}^{+}$$

Throughout aerobic incubation, the reduced Cu and Ti ions were oxidized via oxygen, oxidase enzymes, and oxidizing biomolecules that occupying reaction sphere. Meanwhile, in the presence of OH-, the hydroxide forms of both metals generated as declared in the following equations:

8 $${\text{C}\text{u}}^{+} + 2 {\text{O}\text{H}}^{-} +\text{O}\text{x}\text{i}\text{d}\text{a}\text{s}\text{e} \to \text{C}\text{u} {\left(\text{O}\text{H}\right)}_{2\left(\text{s}\right)} + 2 {\text{H}}_{2}$$

9 $${\text{T}\text{i}}^{3}++3 {\text{O}\text{H}}^{-} + \text{O}\text{x}\text{i}\text{d}\text{a}\text{s}\text{e} \to \text{T}\text{i} {\left(\text{O}\text{H}\right)}_{3\left(\text{s}\right)} + 3 {\text{H}}_{2}\text{O}$$

10 $${\text{T}}^{\text{i}2+} +2{\left(\text{O}\text{H}\right)}^{-} + \text{O}\text{x}\text{i}\text{d}\text{a}\text{s}\text{e} \to \text{T}\text{i}{\left(\text{O}\text{H}\right)}_{2\left(\text{s}\right)} + 2 {\text{H}}_{2}\text{O}$$

However, such hydroxide intermediates were less stable forms and dissociated, by the catalysis of fungal biomolecules, into their corresponding oxide variants, which were the most stable forms [110, 111].

11 $$\text{C}\text{u} {\left(\text{O}\text{H}\right)}_{2} \to \text{C}\text{u}\text{O} + {\text{H}}_{2}\text{O}$$

12 $$\text{T}\text{i}{\left(\text{O}\text{H}\right)}_{3} \to \text{T}\text{i}{\left(\text{O}\text{H}\right)}_{2} + {\text{O}\text{H}}^{-}$$

13 $$\text{T}\text{i}{\left(\text{O}\text{H}\right)}_{2} \to \text{T}\text{i}\text{O}\left(\text{O}\text{H}\right) + {\text{H}}_{2}$$

14 $$\text{T}\text{i}\text{O}\left(\text{O}\text{H}\right) \to {\text{T}\text{i}\text{O}}_{2} + {\text{H}}_{2}\text{O}$$

Accordingly, the bioreduction / biooxidation processes of both metal precursors were attained synchronously generating the first CuO and TiO_2_ nuclei [44]. Such nuclei serve as the seeds or embryos assembled into larger crystals of TiO_2_-NPs that encompass CuO-NPs through electrostatic, coordination, van der Waals, hydrogen binding or even dispersion interactions generating nanocomposites architecture [112]. In fact, the mycologically synthesized CuTiNCs were indigenously functionalized by fungal biomolecules that prevailed in the reaction mixture, acting in such way dual and simultaneous role of reduction and self-functionalization. Hence, considered to be more economic by avoiding additional surface-modification step that is substantially required in physicochemical synthesis approaches to maintain the stabilization of nanomaterials as inferred by [113]. Furthermore, the indigenously functionalized NPs-NCs would mediate facile and selective-targeting purposes, in particular the biological applications such as antimicrobial, anticancer and antioxidant, etc. Evidently, the extracellular synthesis of both NPs-NCs is particularly favorable in terms of large-scale manufacture [114].

### Applications of mycosynthesized NPs-NCs

#### Antimicrobial potency

**Fig. 11:**
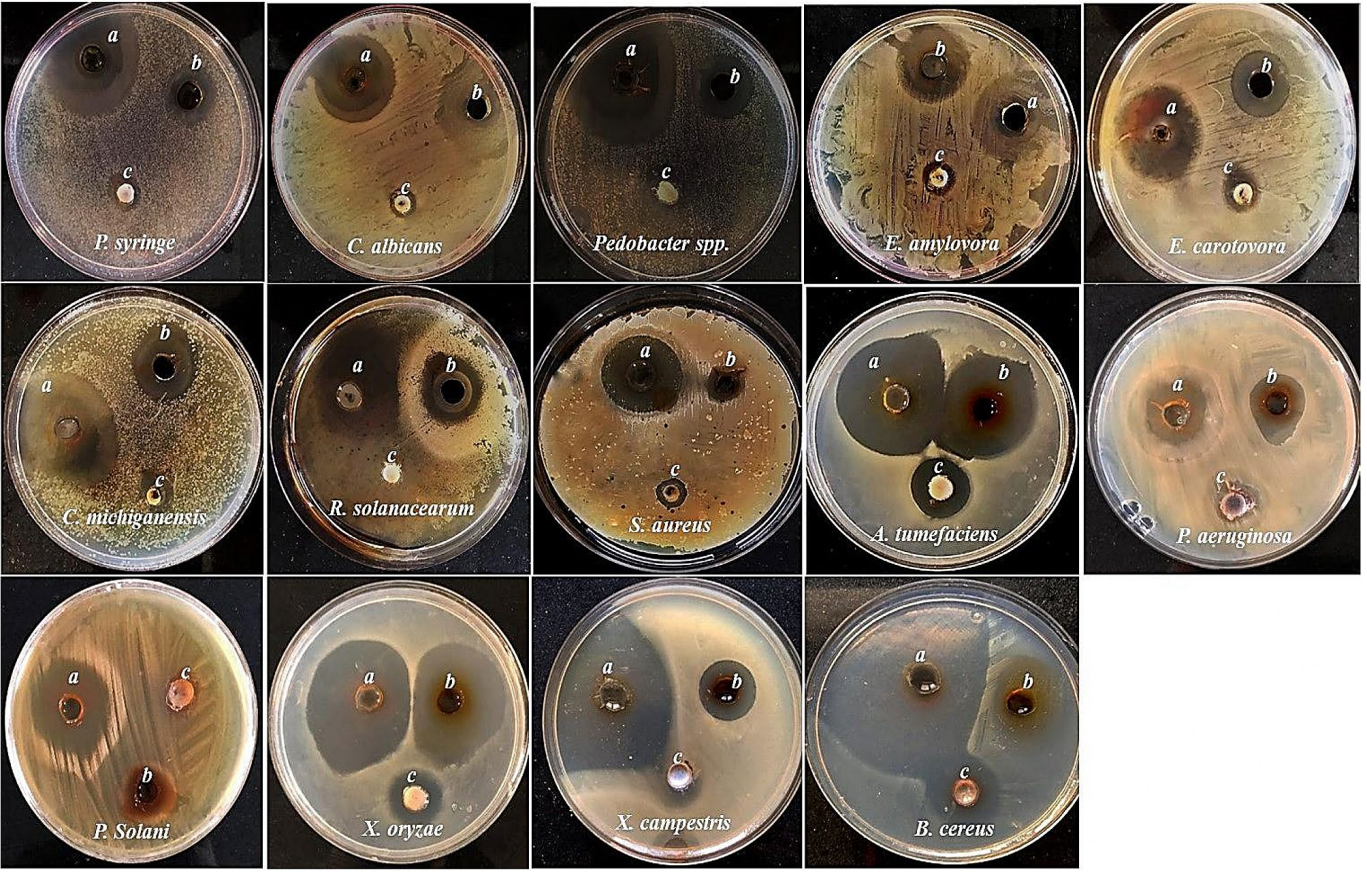
Antimicrobial activity of mycosynthesized CuTiNCs (**a**), CuNPs (**b**) and TiNPs (**c**) against the examined human and plant pathogens by the well diffusion method

Our study focused on assessing the biocide potency of mycosynthesized CuNPs, TiNPs and their hybrid system of CuTiNCs against various pathogenic microbes to cover a wide array of application fields. The assessment included some human pathogens, which are opportunistic and responsible for food intoxication, water-borne diseases, nosocomial and community-acquired infections [115] **.** Besides, the response of phytopathogenic bacteria versus the exposure of mycosynthesized NPs-NCs was also evaluated by ZOI assay (Table 2; Fig. 11). Strikingly, the examined pathogens cause a number of diseases in different plant organs (i.e., fruit, stem, leaf, etc.) such as fruit spots, blackleg, cankers, blights, wilts, soft rots and tumors, leading to seasonal devastation of various crops like potato, tomato, celery, carrot, lettuce, onion, cabbage and fruits as well [116, 117].

**Table 2:** Antimicrobial potency of mycosynthesized CuNPs, TiNPs and CuTiNCs against pathogens via well diffusion method, ZOI represented in cm. The results were expressed as mean ± SEM and (*) indicates the statistical significance (P˂ 0.05)

Microorganism		CuNPs	TiNPs	CuTiNCs
**Human Pathogens**	*B. cereus*	1.9 ± 0.1*	0.5 ± 0.1	2.7 ± 0.1*
	*S. aureus*	1.25 ± 0.05*	0.3 ± 0.05	1.65 ± 0.15*
	*P. aeruginosa*	0.8 ± 0.1	0.2 ± 0.00	1.15 ± 0.15
	*C. albicans*	0.5 ± 0.1	0.1 ± 0.05	0.9 ± 0.1
**Plant Pathogens**	*E. carotovora*	1.6 ± 0.1*	0.2 ± 0.05	1.7* ± 0.1
	*P. syringe*	1.4 ± 0.1*	0.25 ± 0.25	1.85 ± 0.15*
	*P. Solani*	1.35 ± 0.05*	0.15 ± 0.05	1.7 ± 0.2*
	*E. amylovora*	1.1 ± 0.1	0.2 ± 0.1	1.7 ± 0.1*
	*Pedobacter spp.*	1.3 ± 0.1*	0.15 ± 0.05	1.85 ± 0.05*
	*X. oryzae*	1.65 ± 0.15*	0.2 ± 0.1	2.1 ± 0.1*
	*X. campestris*	1.9 ± 0.15*	0.4 ± 0.2	2.15 ± 0.1*
	*A. tumefaciens*	1.55 ± 0.15*	0.2 ± 0.1	1.95 ± 0.05*
	*R. solanacearum*	1.2 ± 0.1	0.1 ± 0.1	1.6 ± 0.2*
	*C. michiganensis*	1.6 ± 0.15*	0.35 ± 0.15	1.8 ± 0.2*

As observed, the capability of NPs-NCs to thwart microbial growth varied significantly. Wherein, the CuTiNCs displayed the superior significant (P ˂ 0.05) antagonistic activity than that showed by their solitary counterparts, as deduced from statistical analysis of the data with one-way ANOVA. Besides, the antimicrobial activity of CuNPs was much significantly higher than that exhibited by TiNPs in all examined pathogens. Arguably, *B. cereus* was the most susceptible pathogen in its response to NPs-NCs among the other examined pathogens. On the other hand, *C. albicans* showed vividly higher resistance. Regarding this, many possible interpretations could be encountered starting with the rapprochement of the genus used, in our study for the synthesis, with the examined strain, reflecting its ability to sequester NPs-NCs or utilize the same metabolic pathway to avoid their toxicity. Let alone the rigid fungal cell wall, which is composed mainly of glucans, chitin and chitosan conjugated with glycosylated protein that triggers them less permeable to toxic materials [118]. Likewise, earlier studies by [63] and [65] accentuated the higher susceptibility of bacteria to NPs treatment than fungi and yeast. Meanwhile, the results of the current study were similar to those obtained by [119, 120] and relatively comparable to those reported by [83].

Despite the disparity in cell wall compositional organization and its structural architecture among all examined pathogens, its role in realizing the differences in microbial sensitivity to the examined NPs-NCs could be overlooked. The examined phytopathogens, which are all Gram-negative, exhibited sensitivity patterns similar to those of Gram-positive. However, the microbial physiology, metabolic performance, and uptake/regulation systems are deemed to be intrinsic factors in managing the resistance and sensitivity profiles among inter and intra-species of the microbes against any antagonistic agent.

#### Antibiofilm activity

**Fig. 12:**
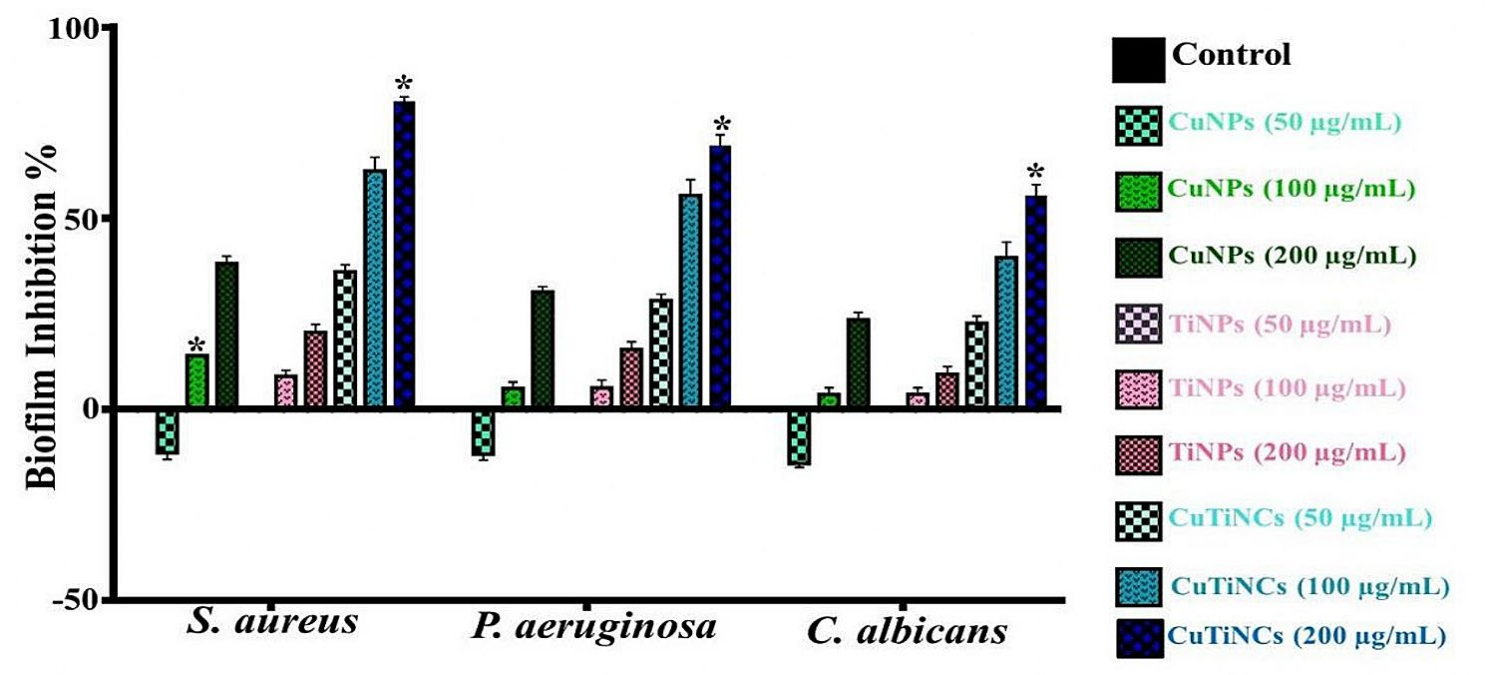
Antibiofilm performance of CuNPs, TiNPs and CuTiNCs in different concentrations (50, 100, and 200 µg /mL) against biofilm-producing pathogens

The antagonistic potency of CuNPs, TiNPs and CuTiNCs in eradicating the biofilm growth of *S. aureus*, *P. aeruginosa*, and *C. albicans* was evaluated (Table 3; Fig. 12). As a general observation, there was a noticeably progressive thwart in the biofilm formation for all examined pathogens in a concentration-dependent manner. Surprisingly, the lowest dose of CuNPs showed enhancement in biofilm growth by 11.3 ± 1.7, 11.7 ± 1.5, and 14.2 ± 0.9% of *S. aureus*, *P. aeruginosa*, and *C. albicans*, respectively. Similar outputs were manifested by [121] and [122]; attesting the fact that upon utilizing sub-inhibitory concentrations of methicillin or any other disinfectant can lead to dramatic induction in the biofilm formation of *S. aureus* as a result of up-regulating the genes encoding surface proteins responsible for the biofilm formation process. Besides, the nature of Cu as vital microelement trigger the biofilm-forming microbes, particularly in our study, utilized it in its lowest concentration (50 µg/mL) in regulating physiological processes and various metabolic activities rather than antagonize their biofilm development. In contrast [123], highlighted that 100 ng of CuNPs facilitated the elimination of biofilm at the initial stage of development, making the eradication process easier.

**Table 3:** Antibiofilm activity of CuNPs, TiNPs and CuTiNCs (50, 100, and 200 µg /mL) fabricated by *Candida sp.* against some biofilm-producing pathogens. The results were expressed as mean ± SEM and (*) indicates the statistical significance (P˂ 0.05)

Biofilm type	CuNPs			TiNPs			CuTiNCs		
	50 µg	100 µg	200 µg	50 µg	100 µg	200 µg	50 µg	100 µg	200 µg
***S. aureus***	-11.3 ± 1.7	13.9 ± 0.2*	38.2 ± 1.9	0	8.6 ± 1.5	20.2 ± 2	36.1 ± 1.8	62.4 ± 3.5	80.3 ± 1.4*
***P. aeroginusa***	-11.7 ± 1.5	5.4 ± 1.7	30.7 ± 1.3	0	5.5 ± 1.6	15.6 ± 2	28.4 ± 1.7	55.9 ± 4.1	68.7 ± 3.0*
***C. albicans***	-14.2 ± 0.9	3.8 ± 1.9	23.4 ± 1.8	0	3.9 ± 1.6	9.2 ± 2	22.5 ± 1.8	39.7 ± 4.0	55.7 ± 3.0*

While no inhibition or enhancement performance was recorded upon treatment with TiNPs (50 µg), Otherwise [51] and [124], elucidated that the utilization of 100 µg/ml of TiNPs was enough to clearly reduce the biofilm formation in *P. aeruginosa* as compared to *S. aureus*. Such variability in the antagonistic performance among different scholars could be ascribed to discrepancies in NPs characteristics (e.g., size, crystal quality, surface area, stability, surface properties, etc.), the synthesis method, and the applied dosage [125, 126]. However, microbial cell physiological properties, cell age, cell surface traits, and microbial load also should be taken into consideration [124, 127], and [65]. Additionally, the whole conditions of the inactivation reaction, such as contact time, organic/inorganic nutrient content, ionic strength, etc., were reported as categorical parameters that manage the NPs-effectiveness in delaying or accelerating the prohibition [128, 129]. Hence, it is important to consider a balance for achieving effective biocide activity without causing unwanted side effects, as recommended by [26] and [130].

Strikingly, in the present investigation, there were common symptoms shared between biofilm-forming pathogens and the examined pathogens, which are dwelling in free-floating or planktonic forms. Firstly, *S. aureus* biofilm exhibited higher sensitivity for the treatments of CuNPs, TiNPs, and CuTiNCs in all examined concentrations. Secondly, the biofilm of *C. albicans* appeared to be the least susceptible to all examined concentrations of NPs-NCs. Thirdly, all concentrations of CuTiNCs displayed a prominent prohibition pattern in all tested biofilm-forming microbes. Notably, a significant inhibition (P ˂ 0.05) for *S. aureus, P. aeruginosa*, and *C. albicans* biofilm development was recorded at 80.3 ± 1.4, 68.7 ± 3.0, and 55.7 ± 3.0%, respectively, upon treatment with 200 µg of CuTiNCs, as testified by ANOVA. In comparison, a study conducted by [44] revealed that 100 mg/mL of CuFe nanocomposites devastated *E. coli* and *S. aureus* biofilm growth by 12 and 49%, respectively; reflecting the potency of the current CuTiNCs in defeating biofilm growth in minuscule amounts. Interestingly, no study examined the antibiofilm potential of the CuTi hybrid.

Based on the previous backdrop, the whole scenario for the antagonistic potentiality of NPs-NCs, under study against various pathogens in either planktonic or biofilm phases, could be envisaged. As outlined by [50] and [131] the strategies followed by NPs in inducing their toxicity could be described as nonspecific, complex, and complicated modes of action. By serving as nanoknives, NPs-NCs commence their action by physical cracking in the cell wall, phospholipid peroxidation, depolymerization of polysaccharides, and concurrent enfeeblement of membrane integrity. All these features collectively result in leakage of cellular components (proteins, lipopolysaccharides, reducing sugars, etc.), dissipation of electron motive force, osmotic imbalance, and a diminishment in ATP intracellular levels. Once NPs, which are less than 80 nm, are sequestered in the cell internally, more destructive effects are exerted, including frustration of biochemical activities and hindering metabolic pathways. Such could be implemented by the higher affinity of metal ions released from NPs-NCs to bind with the thiol group (R − SH) of amino acids, forming (–S–S–) bonds, which deform protein structure, block their active sites, and eventually cause proteins malfunctioning. Let alone their ability to interact with nucleic acids, disrupting, in such a way, replication, DNA repair and protein synthesis processes. However, the intrinsic reason for the overall inhibition lies behind the massive oxidative stress owing to the intense generation of oxygen-free radicals or reactive oxygen species (ROS) like singlet oxygen (^1^O_2_), hydroxyl radicals (OH−) and superoxide radicals (O_2_^−^) via Fenton and Haber–Weiss reactions. Such elevation of ROS intensifies the damage to microbial biomolecules, which terminates metabolic activities and ultimately cell death. Additionally, regarding the antibiofilm potency, NPs-NCs controlled the development of biofilm by limiting the productivity of EPS, prohibiting cell adhesion by modifying surface characteristics and enhancing the quorum-quenching activity of sessile cells.

Worthwhile, the biocide potency of CuNPs exceeded unequivocally that displayed by TiNPs, which is attributed to their higher surface area (surface/volume smaller ratio) of ultrafine-size CuNPs with homogenous dispersity and limited aggregation. All those features allow a faster elution rate of copper ions, more contact with microbial cells, and consequently a higher rate of cytotoxicity. Whereas, the exposure of microorganisms to CuTi in their composite nanoform declared distinct functionalities and an eminent magnitude of toxicity as compared to their sole NPs. That could be ascribed to the synergistic leverage of two different metal oxides rather than merely additive impact, which was consistent with that reported by several research groups [65, 132], and [44]. Intriguingly, the dual exposure of microbial cells to different metal ions simultaneously released from nanomaterials with binary structures put the cells in a sudden shock situation, triggering them handicapped to induce multiple gene mutations for tolerating such synchronized, multiple, and condensed antagonistic doses. Therefore, it is plausible to propose that CuTiNCs exerted their hostility by targeting multiple cellular locations simultaneously with a multimode of action. In the same context [44] and [129], explicated similar findings with CuFeNCs and CuZnNCs, respectively.

Out of the preceding results, the efficacy of mycosynthesized CuTiNCs in ceasing microbial growth and corrupting biofilm formation encouraged their application as an antimicrofouling agent and disinfectant in purifying environmental effluents from their microbial load.

#### Anti-microfouling activity

Biofouling, as an economic and ecological problem, is detected in industrial aquatic processes, food and beverage industries, water desalination systems, oil industry grates, cooling systems, electric cables, and pipelines of water treatment, storage, and distribution. It involves the accumulation of organic materials such as polysaccharides, proteins, and glycoproteins, followed by the colonization of biofilm-forming organisms (e.g., bacteria, fungi, phytoplankton, algae, and protozoa) on submerged surfaces. Such microfoulers represent the main focal point and primary key step in establishing a rigid three-dimensional polymeric matrix that captures larger organisms of macrofoulers (e.g., macroalgae, bryozoans, mussels, barnacles, and tube worms). Economically, to reduce energy consumption, maintain wet surfaces of pipelines, and protect the environment from pathogen dissemination, the development of antifouling coating or painting is the decipher [133]. Worthwhile, organotin (tributyltin) and heavy metal-based paints are the most often employed effective biocide, Nevertheless, owing to environmental damage caused by their broad spectrum cytotoxicity (in ppb) against target and nontarget organisms, their applications became limited [134]. Therefore, the utilization of inorganic NPs as main constituents of antifouling agents has gained momentum.

**Fig. 13:**
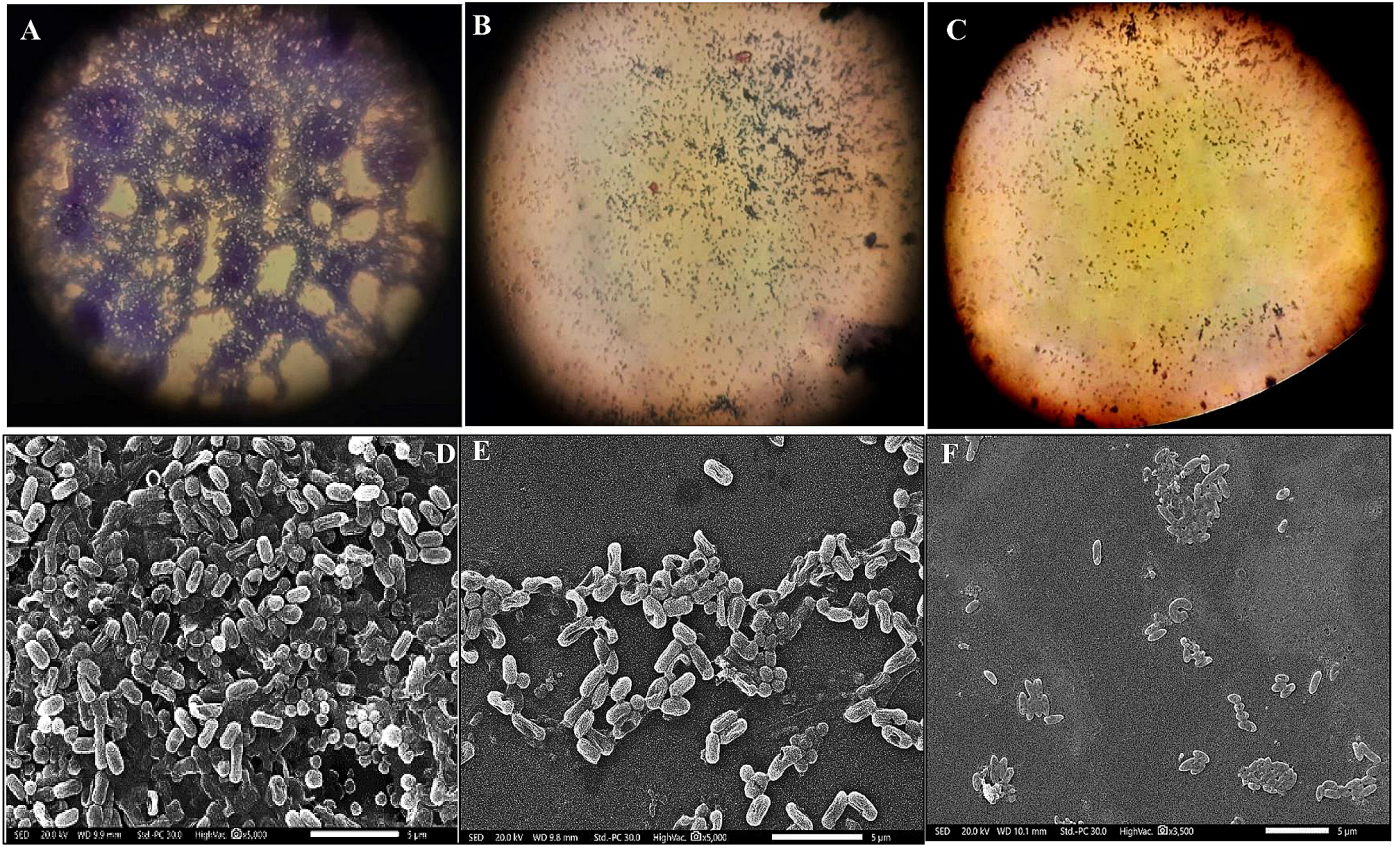
Anti-microfouling potentiality of CuTiNCs via light microscope (**A, B**, and **C**) and SEM (**D, E**, and **F**) of control, and treatment at 100 µg/ml and 200 µg/ml, respectively

Herein, CuTiNCs showed significantly (P ˂ 0.05) distinguished anti-microfouling properties by lessening the density and diversity of adhered marine biofilm-forming microbes by 64.63 ± 3.5, and 89.82 ± 4.3% for 100 and 200 µg/ml, respectively. The images of the light microscope demonstrated that the surface area of the control uncoated slide was harbored with an evenly dispersed confluent, prolific, and dense biofouling load. In comparison, the treated slides appeared to have fewer cells dwelling on the coated surface in a separate manner. For more morphological changes, SEM was employed. (Fig. 13) demonstrated the destructive potentiality of CuTiNCs against marine microfoulers more deeply than a light microscope. Wherein, multilayer aggregations of rods and coccid-shaped cells appeared compactly packed and immersed in a dense EPS matrix in the untreated control sample. However, a few numbers of microfouler bacteria were loosely scattered, reflecting the decay of the EPS skeleton. Besides, treatment with CuTiNCs resulted in dramatic deformation of the cells, represented by the presence of wide furrows, implying cell membrane deterioration and disability to colonize CuTiNCs-coated slides and construct their EPS lattice. Both images of light and SEM emphasize the potential of CuTiNCs in inhibiting microfouler settlement. Recent studies documented the incorporation of CuONPs and TiO_2_NPs in fouling polymeric membranes to consolidate their hydrophilicity and antimicrobial behavior [135, 136], and [137]. Hereby, the antagonistic feature of CuTiNCs impeded the biofilm biomass coverage and disrupted its architecture and distribution, which is a prerequisite step in the whole biological fouling process, as declared by [138] and [53]. It is important to mention that the capability of CuTiNCs to prevent macrofouling development in *in-situ* fields will be implemented in an ongoing study. Hence, the current study succeeded in finding an alternative solution for the micro-fouling problem through recruiting CuTiNCs as antifouling paints.

#### Environmental effluents disinfection

Microbial pollution of aquatic environments menaces the sanitary state of water bodies, which consequently influences negatively the quality of water specified for drinking and irrigation, in particular with the continuous uplifting in population. The discharges from decontamination stations, hospitals, domesticated animals, industries, and water treatment plants are considered to be the major sources for microbial contamination [88, 139]. The arrival of such contaminated sources into the drinking water is a real peril that touches public health, which may result in the dissemination of epidemics and the fall of a country’s economy. Therefore, there is an urgent demand for recruiting proper disinfection practices for purifying contaminated water until it reaches standard limits for consumption, especially given the current situation of the water crisis. On the account of several limitations regarding the chlorination approach, which is the most effective and commonly applied disinfection method, the endeavors for alternative green means are continuously implemented. Accordingly, recent literature has devoted different NPs-NCs to this concern. Meanwhile, based on international standards of environmental guidelines, there are some criteria, such as total plate count (TPC), mold & yeast (TMY), and indicator organisms, including total coliforms (TCs) and fecal *Streptococcus* (FS), that should be determined, and their concentration shouldn’t exceed a certain allowable limit to be safely utilized [140]. Hence, in our study, the disinfection potency of CuTiNCs (100 and 200 µg/ml) was scrutinized in treating two real effluent samples through defining TPC, TMY, TCs, and FS.

As observed in Table (4), the enhancement in microbial inhibitory power was associated with an increase in the treatment dose. Virtually all the examined parameters of TPC, TM, TCs, and FS revealed the distinct disinfection potentiality of CuTiNCs in controlling and inhabiting the microbial content in domestic and agricultural effluents. Wherein, 100 and 200 µg/ml of CuTiNCs engendered about 68.23 and 84.48% reductions in the TPC content of domestic effluent and 55.95 and 73.94% for agricultural effluent, respectively. Whereas, the suppression of mold and yeast content induced by both doses recorded 59.81 and 78.69% for domestic effluent and 47.40 and 67.93% for agricultural effluent, respectively. Regarding indicator microorganisms, the disinfection potency reached 90.43 and 75.57% for TCs in domestic and agricultural effluents, 98.11% and complete elimination of FS, respectively, upon treatment with 200 µg/ml of CuTiNCs. Generally, CuTiNCs exerted a higher disinfection potentiality in domestic effluent than that observed in agricultural sample. The presence of heavy metals and residues from pesticides, herbicides, and fertilizers was proposed to induce a greater tolerance modality for the agriculture-dwelling microbiota than that in municipal wastewater, which was enriched with residues of organic nutrients as accentuated formerly by water quality studies of physicochemical parameters [55]. Additionally, the presence of a higher content of dissolved solids and cations, such as Ca^2+^ could reveal intricacies in the interaction between NCs and the microbial surface. That could happen through the adsorption of such cations on negatively charged CuTiNCs, neutralizing their surface and generating large floccules, which subsequently hampered the antagonistic effect of the released CuO and TiO_2_ ions [138]. Therefore, the physicochemical properties of wastewater are an incontestably conclusive factor in determining the adequate dose required for implementing an acceptable disinfection process. Eventually, the collective traits of mycologically synthesized /functionalized CuTiNCs and the synergism provided by both ions released from the hybrid NCs will open up innovative avenues for their recruitment in various technological applications.

**Table 4:** Disinfection potentiality of 100 and 200 µg/ml of CuTiNCs in decontaminating domestic and agricultural effluents from some microbial parameters. The results were expressed as mean ± SEM and (*) indicates the statistical significance (P˂ 0.05)

Parameter	CuTiNCs Dose (µg/ml)	Domestic effluent Count (CFU/mL)		Agricultural effluent Count (CFU/mL)	
		Control	Treated	Control	Treated
**Total Plate Count**	100	1.42 × 10^7^ ± 2.95 × 10^4^	4.51 × 10^6^ ± 3.5 × 10^5*^	4.09 × 10^5^ ± 1.2 × 10^2^	1.80 × 10^5^ ± 8.85 × 10^3*^
	200		2.20 × 10^6^ ± 1.08 × 10^5^		1.065 × 10^5^ ± 7.83 × 10^3*^
**Mold &Yeast**	100	1.25 × 10^5^ ± 8.65 × 10^3^	5.06 × 10^4^ ± 8.65 × 10^3^	1.63 × 10^4^ ± 2.19 × 10^3^	8.58 × 10^3^ ± 9.16 × 10^2^
	200		2.68 × 10^4^ ± 2.77 × 10^3^		5.23 × 10^3^ ± 2.9 × 10^2^
**Coliforms**	100	1.58 × 10^4^ ± 1.06 × 10^2^	5.83 × 10^4^ ± 6.78 × 10^2^	1.089 × 10^4^ ± 3.37 × 10^2^	5.33 × 10^3*^ ± 2.05 × 10^2*^
	200		1.51 × 10^4^ ± 1.06 × 10^2^		2.66 × 10^3^ ± 1.39 × 10^2^
**Fecal** ***Streptococcus***	100	1.06 × 10^2^ ± 12	40 ± 2.5	41 ± 1	8 ± 2
	200		2 ± 1*		0*

## Conclusion

To summarize, this investigation, for the first time, accentuates the ability of *Candida sp.* towards the fabrication of binary hybridized nanoforms of CuTiNCs. Through a simple eco-friendly bottom-up approach, the bionanofactory *Candida sp.* was challenged for both extracellular and intracellular production of CuNPs, TiNPs and their nanocomposites. The mycofabrication of CuNPs, TiNPs, and CuTiNCs was assured via TEM, SEM, XRD, UV-Vis spectroscopy, FTIR, EDX, ζ-potential, and TGA. The biocidal activity of myco-synthesized NPs-NCs was assessed against a vast array of human as well as plant pathogens. Besides, the antibiofilm activity of CuNPs, TiNPs, and CuTiNCs in different concentrations was also defined. A significant inhibition (P ˂ 0.05) exerted by CuTiNCs (200 µg/mL) reached to 80.3 ± 1.4, 68.7 ± 3.0, and 55.7 ± 3.0% in defeating *S. aureus, P. aeruginosa*, and *C. albicans* biofilms development, respectively. The results unveiled the superior potency of CuTiNCs compared to their single nanoforms, in a dose-dependent modality, which favored their applications in wastewater treatment as antifouling agents and disinfectants. The light microscopy and SEM depicted the capability of 200 µg/ml CuTiNCs in inhibiting microfouler settlement by 89.82 ± 4.3%, while deteriorating EPS architecture and cell morphology. However, 100 and 200 µg/ml of CuTiNCs exhibited an eminent disinfection potential and diminished the microbial load of bacteria, molds, yeast, and indicator organisms of coliforms and fecal *Streptococci* after 2 h of exposure. Interestingly, the mutual corporative interactions of Cu and Ti ions from the CuTiNCs spark immense interest to be invested in prospective medical and energy-mediated applications.

## Data Availability

No datasets were generated or analysed during the current study.
